# Spots, stripes, and spiral waves in models for static and motile cells: GTPase patterns in cells

**DOI:** 10.1007/s00285-021-01550-0

**Published:** 2021-03-04

**Authors:** Yue Liu, Elisabeth G. Rens, Leah Edelstein-Keshet

**Affiliations:** 1 Department of Mathematics, University of British Columbia, Vancouver, V6T 1Z2 BC Canada; 2 Present Address: Mathematical Institute, University of Oxford, Oxford, OX2 6GG UK; 3 Present Address: Delft Institute of Applied Mathematics, Delft University of Technology, Delft, The Netherlands

**Keywords:** Pattern formation, Intracellular signaling, GTPase, wave-pinning, Local perturbation analysis, Static and moving boundary computation, 92C15, 92C37

## Abstract

The polarization and motility of eukaryotic cells depends on assembly and contraction of the actin cytoskeleton and its regulation by proteins called GTPases. The activity of GTPases causes assembly of filamentous actin (by GTPases Cdc42, Rac), resulting in protrusion of the cell edge. Mathematical models for GTPase dynamics address the spontaneous formation of patterns and nonuniform spatial distributions of such proteins in the cell. Here we revisit the wave-pinning model for GTPase-induced cell polarization, together with a number of extensions proposed in the literature. These include introduction of sources and sinks of active and inactive GTPase (by the group of A. Champneys), and negative feedback from F-actin to GTPase activity. We discuss these extensions singly and in combination, in 1D, and 2D static domains. We then show how the patterns that form (spots, waves, and spirals) interact with cell boundaries to create a variety of interesting and dynamic cell shapes and motion.

## Introduction

The dynamics of the actin cytoskeleton determines internal cell structure, cell shape, and cell motility. By accumulating at a cell edge, filamentous actin (F-actin) produces outwards protrusion. Actin assembly is regulated by signaling networks. Central in those networks are the small GTPases, Rac, Cdc42, and Rho. Rac promotes assembly of F-actin, whereas Rho activates myosin motors. The interactions of Rac, Rho, Cdc42, and other molecular players has been modeled in previous work (Mori et al. 2008; Verschueren and Champneys 2017; Holmes et al. 2012a; Holmes and Edelstein-Keshet 2016; Zmurchok et al. 2018; Walther et al. 2012; Edelstein-Keshet et al. 2013; Jilkine and Edelstein-Keshet 2011; Otsuji et al. 2007) both in 1D and 2D. These studies made different modelling decisions and ranged from simple (Mori et al. 2008) to detailed (Marée et al. 2008). It is challenging to determine parameter sensitivity and map out regimes of behavior of the more detailed models. This motivates studying minimal models that showcase the possible realms of predicted behavior.

It was shown previously that the biology of GTPases permits a single member of this family to spontaneously polarize (i.e. form spatial regions of high vs low activity). This idea was the basis of the wave-pinning model (Mori et al. 2008, 2011), and depends on the large difference in diffusion of the active (slow) and inactive(fast) forms of a GTPase.

Several models have been examined mathematically to describe how a single GTPase coupled to other effectors or influences could result in spatio-temporal patterns. These include a GTPase with sources and sinks (Verschueren and Champneys 2017, see also Champneys et al. 2021), with feedback from F-actin (Holmes et al. 2012a; Mata et al. 2013), with mechanical tension (Zmurchok et al. 2018) and with effects of changing cell size (Buttenschön et al. 2019). Many of these were explored in reaction-diffusion (RD) equations within a 1D static single cell domain or with spatially uniform distribution in each of many cells (Zmurchok et al. 2018). Some of the behaviors found in such models include, traveling waves, pulses, or oscillating fronts (Holmes et al. 2012a; Mata et al. 2013), or localized peaks and “solitons” (Verschueren and Champneys 2017).

Here we have three main purposes: (1) to explore what happens when two distinct minimal models are coupled, and whether this leads to new behavior, (2) to study these systems in 2D domains to determine whether they produce spots or stripes, and (3) to simulate the same models on a deforming 2D domain depicting the shape and motility of a cell.

**Fig. 1:**
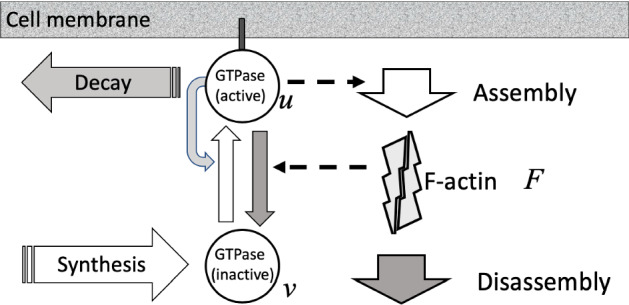
Schematic diagram of the models. The original wave-pinning model consists of GTPase (circles) in the active, (membrane-bound) form, *u* and inactive form *v*, with positive feedback (curved grey arrow) from *u* to its own activation (upwards white arrow), forming a positive feedback loop. The F-actin extension model (Holmes et al. 2012a) includes GTPase activation of F-actin assembly and GTPase inactivation by F-actin (dashed arrow), forming a negative feedback loop. The source-sink (nonconservative) extension by Verschueren and Champneys (2017) includes removal of active GTPase and synthesis of inactive GTPase so that the total amount is no longer conserved

Biological motivation for this work comes from several sources. (A) Waves of actin are observed in a number of experimental systems (Inagaki and Katsuno 2017). In some of these, such waves are seen to cause cell edge to cyclically protrude outwards (as the waves impinge on the cell edges). We wondered whether a model for Rac interacting with F-actin could mimic this kind of behavior. (B) The GTPase model generalized by the group of Alan Champneys in Verschueren and Champneys (2017) converts the polarizing cell behaviour into multiple coexisting peaks. We wondered how such peaks would interact with cell boundaries, and, in particular, whether they would be associated with smaller protrusions such as filopodia. (C) In some cells, notably the embryos of C. elegans, localized Rho-associated actin clusters are seen to “blink” (oscillate temporally while maintaining a fixed location) (Robin et al. 2016). We asked whether the combined F-actin-Rho model with localized sources could account for such behavior. A schematic description of the model is provided in (Fig. 1).

We first briefly review the three classes of minimal models, show results for the combined model, and then demonstrate the novel 2D behaviors that are observed once these models are simulated in the deforming 2D cell.

## The models

Our model is a system of reaction-diffusion partial differential equations (PDEs) based on the wave pinning model first proposed by Mori et al. (2008). The model is extended with a source and sink terms following Verschueren and Champneys (2017), and feedback from actin, proposed by Holmes et al. (2012a).

### Model equations

The dimensionless form of the model combining both extensions can be written as:

1a $$\begin{aligned} \frac{\partial u}{\partial t}&= \delta \nabla ^2 u +f(u,v,F) - c\theta u , \end{aligned}$$

1b $$\begin{aligned} \frac{\partial v}{\partial t}&= \nabla ^2 v -f(u,v,F) +c \alpha , \end{aligned}$$

1c $$\begin{aligned} \frac{\partial F}{\partial t}&= \epsilon (k_n u - k_s F) , \end{aligned}$$

1d $$\begin{aligned} f(u,v,F)&=A(u) v - \left( \eta + s \frac{F}{1+F} \right) u , \quad A(u)= k + \gamma \frac{u^n}{1 + u^n},\\ \frac{\partial u}{\partial \mathbf {n}} \bigg |_{\partial \varOmega }&= 0, \quad \frac{\partial v}{\partial \mathbf {n}} \bigg |_{\partial \varOmega } = 0, \quad x \in \varOmega , \quad t \ge 0.\nonumber \end{aligned}$$

Here *u*(*x*, *t*) and *v*(*x*, *t*) represent the active and inactive GTPase, respectively. *F*(*x*, *t*) represents filamentous actin (F-actin). $\delta \ll 1$ is the diffusion coefficient for the active form, which is slow due to attachment to the membrane. The reaction function *f*(*u*, *v*, *F*) describes the net rate of GTPase activation, with *A*(*u*) representing activation rate. Parameters $k, \gamma , \eta , s$ are the basal activation rate, self-feedback activation, basal inactivation and actin-feedback inactivation rates, respectively. The parameters $\alpha$ and $\theta$ were introduced by Verschueren and Champneys (2017) to break conservation. These rates reflect, respectively, the degradation of GTPase when it is in an active form, and the de novo synthesis of inactive GTPase. Neumann boundary conditions are used to represent the fact that GTPases and F-actin do not leak out of the cell edges.

Setting $c=s=0$ reduces the system to the original wave pinning (WP) model (Mori et al. 2008), which conserves the total $u+v$ inside the domain. The model has been analyzed in detail elsewhere (Mori et al. 2011), but we briefly mention its key property: under specific parameter settings, the WP model sustains waves that decelerate and stall in the domain, leading to a stable spatially heterogeneous steady state distribution of *u* (the “pinned wave”).

When $c=1, s=0$, the system corresponds to the non-conservative (NC) model of Verschueren and Champneys (2017). When $c=0, s>0$, we have the actin feedback (AF) model of Holmes et al. (2012a). While each of the above models has been studied previously, here we will also be concerned with their union, i.e. the so-called “combined model” (CM) with $c=1, s>0$. The four models of interest are then (I) WP, (II) NC, (III) AF, and (IV) CM. These four models all have very distinct characteristic behaviors. We will consider these models in several settings (A) a 1D spatial domain, as previously described in the literature, (B) a static 2D spatial domain where we can distinguish between spots and stripes, and finally (C) a deforming domain whose boundary dynamics is coupled to the evolving solution *u* (or *F*) of the PDE.

**Fig. 2:**
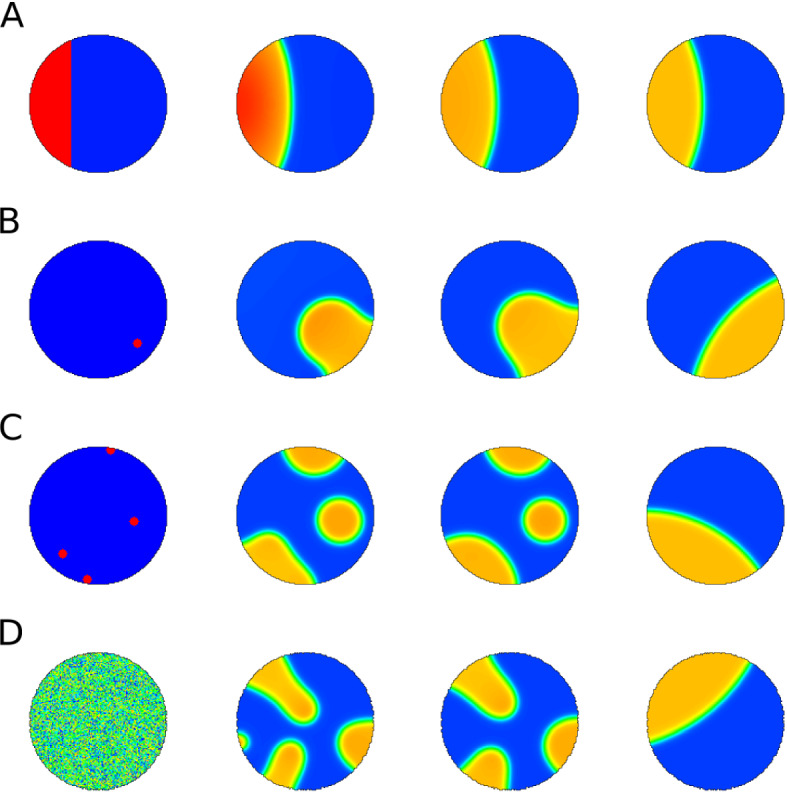
Time sequence (left to right) of *u*(*x*, *y*, *t*) in 2D simulations of the original wave pinning model (Eq. (1)a, b with $s = 0, c = 0$. The circular domain is static, with no-flux boundary conditions and various initial conditions **a**–**d** but the same total amount *w* (see Eq. (3)). **a** stripe of high *u* (red) on the left, **b** randomly placed peak of *u*, **c **four random peaks, **d** uniform random noise. Sequence shows $t=0, 50, 100$ MCS and a later steady state profile. **a** reaches steady state fastest. **c** and **d** take longer as multiple domains have to merge. Ultimately all cases result in a pinned wave at various locations along the cell edge. Parameters are from Table 4 (WP) except $\delta = 0.1$ and $\theta =4.5$

**Fig. 3:**
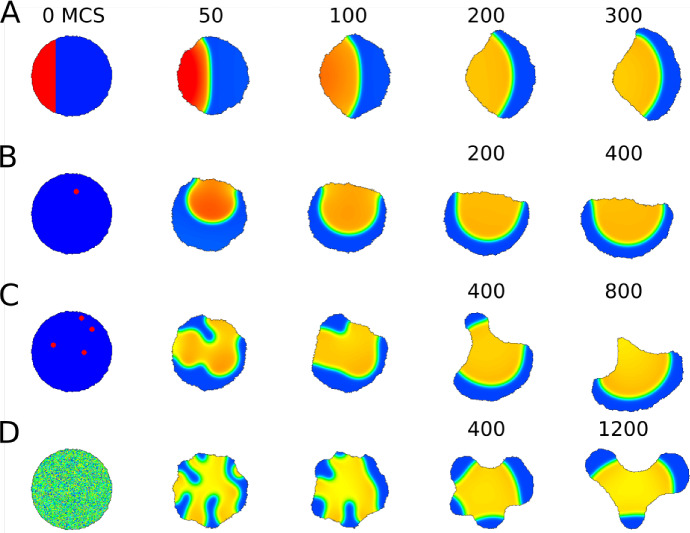
As in Fig. 2, but with boundary deformation that depends on *u*(*x*, *y*, *t*) at the cell edge. Here *u* represents the GTPase Rho, whose activity promotes retraction of the boundary into the domain. **a**, **b** The cell quickly polarizes (wavepinning of *u*) and then moves in a directed fashion (blue denotes low *u* at the front of the cell). **c**, **d**, edge retraction has trapped a plateau of *u* internally; this takes some time to resolve into a pinned wave in **c** or persists for a much longer time in (**d**). Parameters are as in Fig. 2, with CPM Parameters: $a=10000$, $\lambda _a=0.02$, $p=400$, $\lambda _p=0.5$, $J=60$, $r=3,\xi (r)=18$, $\beta =10$, $T=20$

### Domain, geometry, and initial conditions

In many previous papers, simulations were restricted to 1D (Holmes et al. 2012a; Mata et al. 2013), but a variety of actin wave models exist in more detailed geometries, including 2D (Doubrovinski and Kruse 2011) and 3D (Bretschneider et al. 2009).

Most of our analysis and supporting numerical results are given in 1D for simplicity. Ultimately, we aim to show patterns stemming from the full model (1) interacting with deforming 2D domains. Hence, at the outset, we establish a basic “default wave-pinning behaviour” in both static and deforming circular domains for purpose of comparison, as shown in Figs. 2 and 3, respectively. For the deforming (initially circular) domain, we use the Cellular Potts Model (CPM) to simulate a dynamic 2D shape, with PDE solution influencing the boundary motion. The methods, assumptions, and results are introduced in Sect. 5.3. Similar results have been shown previously by Marée et al. (2012) for a more biologically detailed model (and elsewhere for simple wavepinning, e.g. Vanderlei et al. 2011), but Fig. 3 makes for direct comparison with simulations of the full model in Figs 18 – 21 in a later section.

As also demonstrated in the preliminary Figs.  2 and  3, pattern formation can be initiated in many ways, including step function (A), peak(s) placed at random locations (B,C) noisy initial levels of *u* (D) or other distributions (see also (Jilkine and Edelstein-Keshet 2011)).

Further on, we adapt the appropriate ICs to the purpose at hand. In 1D we wish to find major pattern features, such as number or spacing between peaks. In the 2D static simulations, our purpose is to distinguish spots from stripes. In the 2D deforming domains of the full model later on, we are interested in interactins of wave with the boundary motion and so we use initiate conditions that have elevated *u* away from the boundaries.

For the ease of analysis and identification of distinct patterns, we first discuss and examine results in a 1D spatial version of the models. We can interpret this 1D geometry in one of two classic ways: (1) as a cross-section along the diameter of a cell. This cross-section neglects any variation in the cell thickness and includes both the intracellular volume (cytosol) and the top and bottom membranes at every point. Neumann (no flux) boundary conditions are used for the endpoints of the interval. (2) Alternatively, another common assumption is a 1D cell perimeter. In this case, the region considered is close to the cell membrane, with periodic boundary conditions. Here we adhere to the first approach. The case of 1D dynamic cell size is considered in Buttenschön et al. (2020).

## Methods of analysis and computational methods

We briefly describe methods used to analyse the models. We use local perturbation analysis (LPA) to study the bifurcation behavior of each model, and compare with results from Turing Linear stability analysis. A full description of these methods is found in the MSc thesis by one of us (YL) (Liu 2019).

### Local perturbation analysis

Local perturbation analysis is a method for examining the evolution of a localized perturbation to a homogeneous steady state (HSS) for a fast-slow diffusion-reaction system. It provides a way to systematically detect certain forms of nonlinear instabilities that are not detectable by the more traditional Turing analysis. LPA was first used by Grieneisen (2009), and has been used in Edelstein-Keshet et al. (2013); Holmes and Edelstein-Keshet (2016); Holmes et al. (2012a); Mata et al. (2013) and elsewhere to analyze wave pinning and related models.

The basic idea of LPA is to take the limit where the slow diffusion coefficients go to 0 and the fast diffusion coefficients go to infinity. We then consider an initial condition where the system is at HSS with a localized perturbation in the form of a spike of infinitesimal width but finite height. The behavior of the PDE can then be captured with an ODE system with “global variables” representing the levels of the PDE variables away from the spike, and “local variables” for the slow PDE variables at the spike. For example, using subscript *L* to denote local variables, the LPA system for our combined model (1) is:

2a $$\begin{aligned} \frac{\partial u}{\partial t}&= f(u,v,F) - c\theta u , \end{aligned}$$

2b $$\begin{aligned} \frac{\partial v}{\partial t}&= -f(u,v,F) +c \alpha , \end{aligned}$$

2c $$\begin{aligned} \frac{\partial F}{\partial t}&= \epsilon (k_n u - k_s F), \end{aligned}$$

2d $$\begin{aligned} \frac{\partial u_L}{\partial t}&= f(u_L,v,F_L) - c\theta u_L , \end{aligned}$$

2e $$\begin{aligned} \frac{\partial F_L}{\partial t}&= \epsilon (k_n u_L - k_s F_L) . \end{aligned}$$

In the cases where $c=0$ or $s=0$, we will use mass conservation to remove irrelevant equations and eliminate degeneracy. This allow us to easily produce bifurcation diagrams using AUTO (Doedel 1981) and delineate parameter regimes. Notice that the LPA system (2) contains the well-mixed system (i.e. the system without local variables), so any features (branches and bifurcations) of the well-mixed system will also be present in the LPA system. Hence we can obtain any information that can be gained by analyzing the well-mixed system through LPA.

### Bifurcation analysis

We refer to branches of equilibria and periodic solutions in the LPA system that are also present in the well-mixed model as “global” branches, as they correspond to solutions in which the local variables are equal to the global variables and the spike disappears, i.e. a homogeneous solution. The other branches are referred to as “local” branches; they correspond to some kind of pattern.

We classify the parameter regimes into three categories: (a) stable, where only global branches are stable. In this regime, no pattern can arise from localized perturbation; (b) polarizable, where stable global and local branches coexist. In this regime, patterns can form only if the perturbation is sufficiently strong. Finally, (c) unstable, where all global branches are unstable. In this regime even infinitesimal perturbations can lead to pattern formation. In Appendix 8, we show that this is equivalent to the classical Turing regime.

The sets of parameters for each model are listed in Table 4. For the cases where the total amount of GTPase is conserved, we define the total mass of GTPase in the cell,

3 $$\begin{aligned} w = \int _\varOmega (u+v) dx , \end{aligned}$$

as an additional constant parameter. This allow us to eliminate *v* from the equations by writing it in terms of *u* and *w*.

All bifurcation diagrams follow AUTO’s conventions. On one-parameter diagrams, red/black curves indicate positions of stable/unstable equilibria respectively, while green/blue indicate the range of stable/unstable limit cycles. On two-parameter diagrams, red/light blue/dark blue curves trace the position of limit points (fold points)/branch points (transcritical points)/Hopf points, respectively.

### Computational methods in 2D

The numerical methods for static cell shapes are described in Appendix 9. For the circular and deforming cell shapes shown in Figs. 2 and 3 and later, we use the Cellular Potts Model (CPM), described in detail in (Marée et al. 2007) and briefly summarized in a later section and Appendix 10. The essential feature of the CPM is its ability to track an evolving domain shape. The model PDEs are solved on the domain, and the values of the variables at the domain edge affect the retraction (or protrusion) of the edge according to the changing value of CPM Hamiltonian. For example, in Fig. 3, a high value of *u* is assumed to favour local retraction. For Fig. 2, the same computation was performed without allowing the domain to deform.

## Results

We next apply the methods to compare the behaviors of the four models of interest.

### Preliminary wave-pinning simulations

The behaviour of the original WP model is shown on a static and on a deforming domain for several initial conditions (Figs. 2, 3) including a region of elevated *u* on the left part of the disk, a small peak or several peaks of high *u*, or a noisy initial profile of *u* in the disk. The static domain rapidly develops a polarized “pinned” *u* profile. The final patterns largely are qualitatively similar, with one caveat. In Fig. 3d we see that noisy initial conditions can give rise to an internal plateau of *u* that persists for a long time in a non-convex boundary. In that case, the cell develops a few protrusions that coarsen into three lobes.

**Fig. 4:**
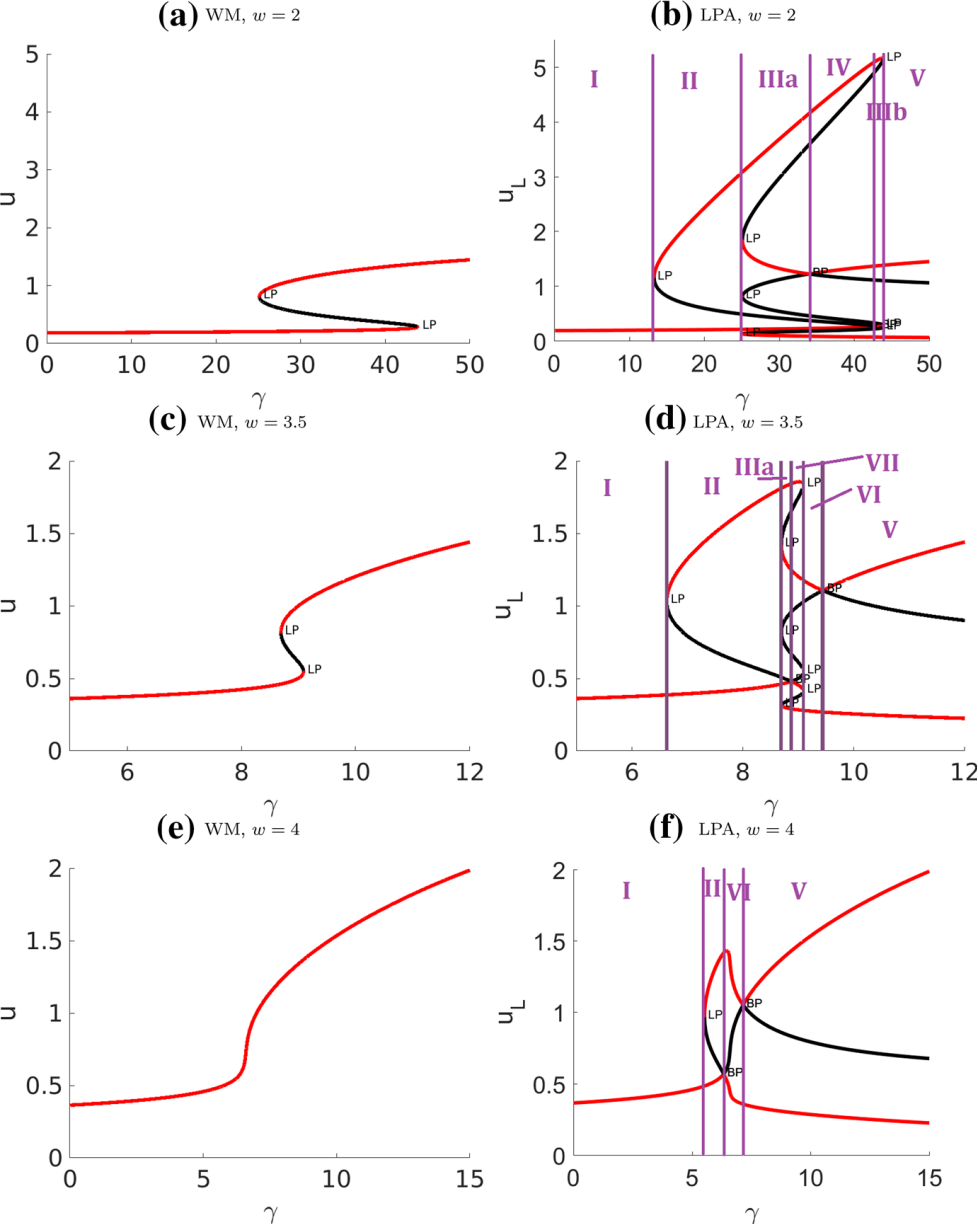
Bifurcation diagrams of the well-mixed (WM) and LPA wave pinning system with respect to the rate of activation parameter $\gamma$. Other parameters as in Table 4 (WP) except *w*. The purple lines are located at bifurcation points separating the distinct regimes. Note that the “global branches” (curves in the WM diagrams) also appear in LPA, though their stability can be different in LPA over certain intervals. The numerical simulation associated with this system is presented in Fig. 9

**Fig. 5:**
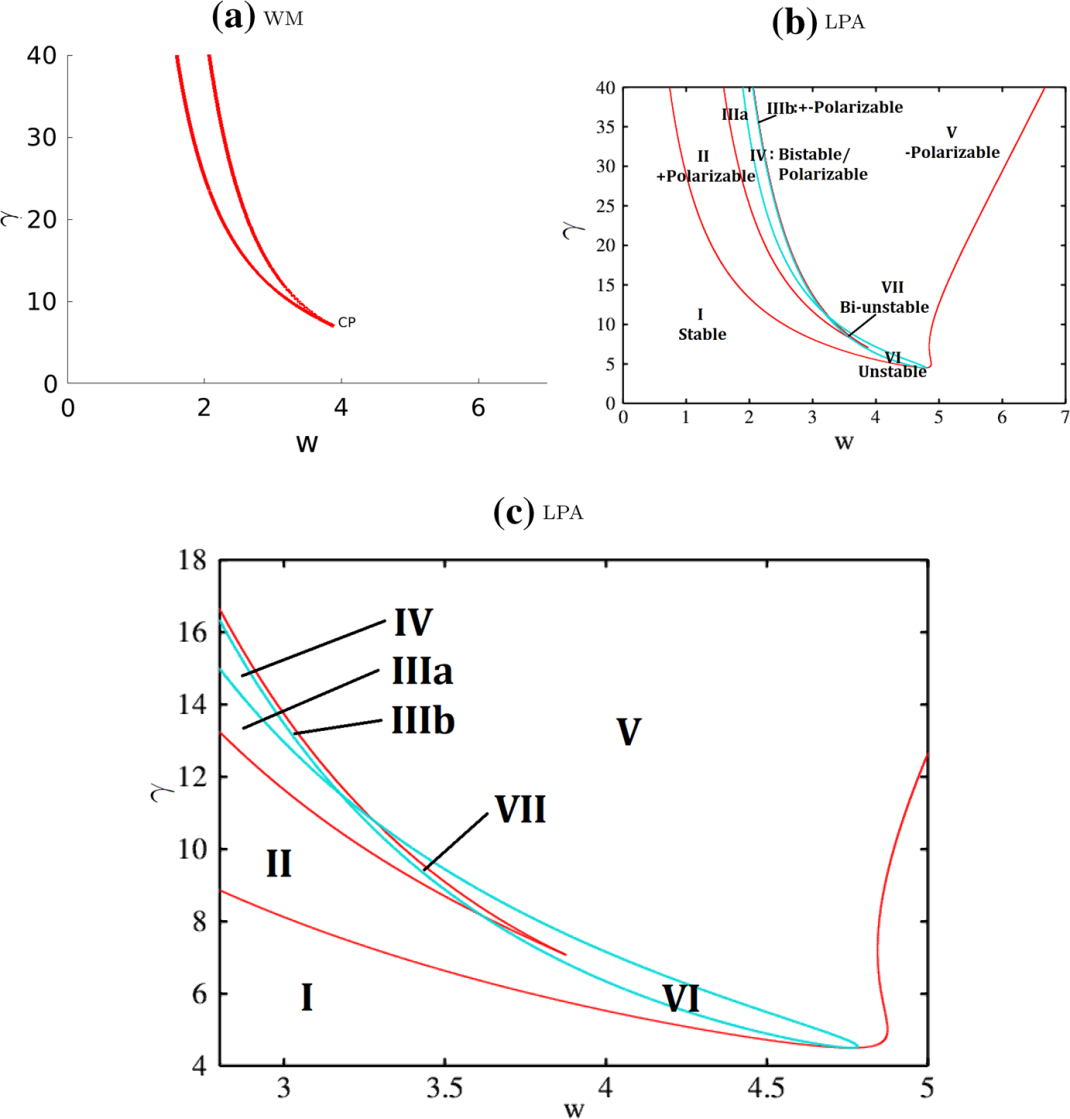
Two-parameter bifurcation plots of the wave pinning (WP) model with respect to parameters $w, \gamma$. **a** Well-Mixed (WM) and **b**, **c** LPA system. Other parameters as in Table 4 (WP). Each curve in these diagrams traces the location of a bifurcation point shown in Fig. 4, and forms the boundary of a parameter regime. The one-parameter bifurcation diagrams in Fig. 4 correspond to vertical cross-sections of the diagrams here. The LPA regimes I - VII match with the regimes in Fig. 4(b, d, f). See summary in Table 1. **c** A zoom into the cusps in (**b**). (Compare (**b**) to LPA Fig. 3a of Holmes and Edelstein-Keshet (2016) for the same model with different parameter values: our figures agree on the (red) fold curves but ours includes an additional transcritical curve (light blue) separating several distinct regimes.)

### Wave pinning (WP) model regimes

Based on extensive previous analysis (Mori et al. 2011, 2008; Holmes and Edelstein-Keshet 2016) we highlight the results in Figs. 4 and  5 merely for comparison with the extended model variants. Distinct regimes are summarized in Table 1. We identify $\gamma$ (the magnitude of the only nonlinear term) and *w* (total concentration) as primary parameters of interest. Extending the earlier study (Holmes and Edelstein-Keshet 2016), we also trace a branch of transcritical bifurcation in two-parameter continuation in Fig. 5. This allows us to identify several new regimes. We verify that LPA predictions in each regime are indeed correct with simulations of the full PDEs.

**Table 1:** Summary of the wave pinning (WP) regimes identified in Fig. 4 and 5

Regime	Classification	Description
I	Stable	One stable GB, no LB
II	Polarizable	One stable GB, one stable LB located above the GB
III	Polarizable	One stable GB, three stable LBs located on both sides of the GB
IV	Polarizable	Two stable GBs, three stable LBs: one above both GBs, one in between, and one below both GBs
V	Polarizable	One stable GB, one stable LB located below the GB
VI	Unstable	The only GB is unstable, two stable LBs located on both sides of the GB
VII	Unstable	Three GBs, all unstable, four stable LBs located on both sides of the GB

*GB global branch, LB local branch, Stable all stable branches are global branches, Polarizable there exist both stable global and local branches, Unstable all global branches are unstable, so some local branches have to be stable*

### Non-conservative (NC) model

In addition the main bifurcation parameter $\gamma$, we also take *c*, the parameter that controls the magnitude of the source/sink terms. This model possesses a unique global equilibrium:

$$\begin{aligned} u_* = \frac{\alpha }{\theta }, \quad \ v_* = \frac{c \alpha + \eta u_*}{A(u_*)}=\frac{c \alpha + \eta u_*}{k + \gamma \frac{u_*^n}{1+u_*^n}} \,. \end{aligned}$$

Any local branches $u_{L*}$ must satisfy $f(u_{L*},v_*)=\theta u_{L*}$. After expanding and some manipulations, we obtain

4 $$\begin{aligned} \frac{A(u_{L*})}{A(u_*)}=\frac{u_{L*}}{u_*}. \end{aligned}$$

Since neither *A*(*u*) nor $u_*$ involve *c* and $\eta$, we conclude that the local branches are independent of these parameters. Furthermore, for $\gamma \ll k$, the LHS of (4) $\approx 1$ so $u_{L*}=u_*$, which means that there is no local branch for small $\gamma$.

We will show that for $\gamma \gg k$, there are always a high and low local branches. The low branch is $u_L \approx 0$, since with $\gamma \rightarrow \infty$ and $u_L =0$, both sides of (4) evaluate to 0. With a bit of further manipulation, we get (in the limit $\gamma \rightarrow \infty$):

$$\begin{aligned} h(u_{L_*}) = h(u_*), \quad \text {where } h(u)=\frac{u^{n-1}}{1+u^n} \,. \end{aligned}$$

The function *h*(*u*) satisfies $h(0)=0=h(u \rightarrow \infty )$, and it has a single peak at $u_{p} \ge 1$ (provided $n \ge 2$). Since we focus on parameters with $\alpha <\theta$, that is $u_* <1$, there exists a point $u_{L*}> u_p > 1$ such that $h(u_{L*})=h(u_*)$, which corresponds to the high local branch.

**Fig. 6:**
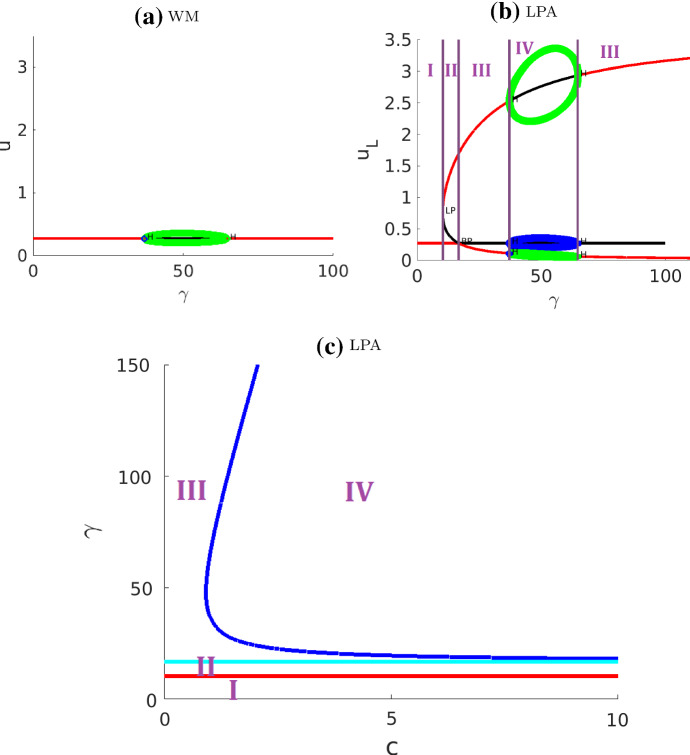
Bifurcation diagrams for the non-conservative (NC) model, with parameter values from Table 4 (CM2) except $\eta =5$. **a** WM, **b**, **c** LPA, using bifurcation parameters **a**, **b** $\gamma$, with $c=1$, **c** *c* and $\gamma$. A thin polarizable regime II is sandwiched between the stable I and Turing III regimes. In the full PDE simulations, which can be found in Figs. 12 and 13 , the triplet of Hopf bifurcations (not present in WP) does not show up as new behavior

In the bifurcation diagrams in Fig. 6, we use parameters from Table 4 (CM2) but with $\eta =5$. (These parameters yield visually optimized bifurcation diagrams whose regimes are neither too wide nor too narrow; the same regimes are present for parameters from Table 4 (NC) used for PDE simulations, but the resulting bifurcation diagram is harder to read.) Fig. 6 identifies four distinct regimes in the LPA system whose interpretation is as in the previous section. The location of the branches agrees with earlier analysis. The regimes are summarized in Table 2.

In Regime I, no pattern forms, as expected. In Regime II a small perturbation decays, but a sufficiently large perturbation will persist. In the full PDEs, such perturbation leads to the soliton solution shown in Fig. 12c,d. Both Regime III and IV are unstable, and any perturbation leads to a Turing-type pattern consisting of a series of evenly spaced, static spikes in the full PDE, as in Fig. 12a,b. The limit cycles in Regime IV suggests that the spikes might oscillate, but this, in fact, does not occur: we found that the PDE behavior is qualitatively indistinguishable in Regime III and IV. This suggests that the Hopf bifurcations in the LPA diagram should be interpreted with caution. We speculate that oscillations predicted by Hopf bifurcations in the local LPA variables could be damped or abrogated by finite diffusion rates in the full PDES. The fact that Hopf bifurcation is a degree 3 phenomenon, whereas fold and transcritical bifurcations are degree 2, might also indicate why LPA may fail to accurately reflect PDE behavior for Hopf bifurcation. This discrepancy points to a limitation of LPA, but a rigorous explanation remains an open problem.

**Table 2:** Summary of the non-conservative (NC) model regimes identified in Fig. 6

Regime	Classification	Description
I	Stable	One stable GB, no LB
II	Polarizable	One stable GB, one stable LB located above the GB
III	Unstable	The only GB is unstable, two LBs located on both sides of the GB
IV	Unstable	One GB, two LBs all unstable, each enclosed by a periodic orbit

Abbreviations as in Table. 1

### Actin feedback (AF) model

We use mass conservation to eliminate *v* from the LPA system as before. The strength of actin feedback *s* and the basal rate of activation *k* were our bifurcation parameters. LPA for this model was previously discussed in Holmes et al. (2012a); Mata et al. (2013), but here we traced more bifurcations in greater detail.

**Fig. 7:**
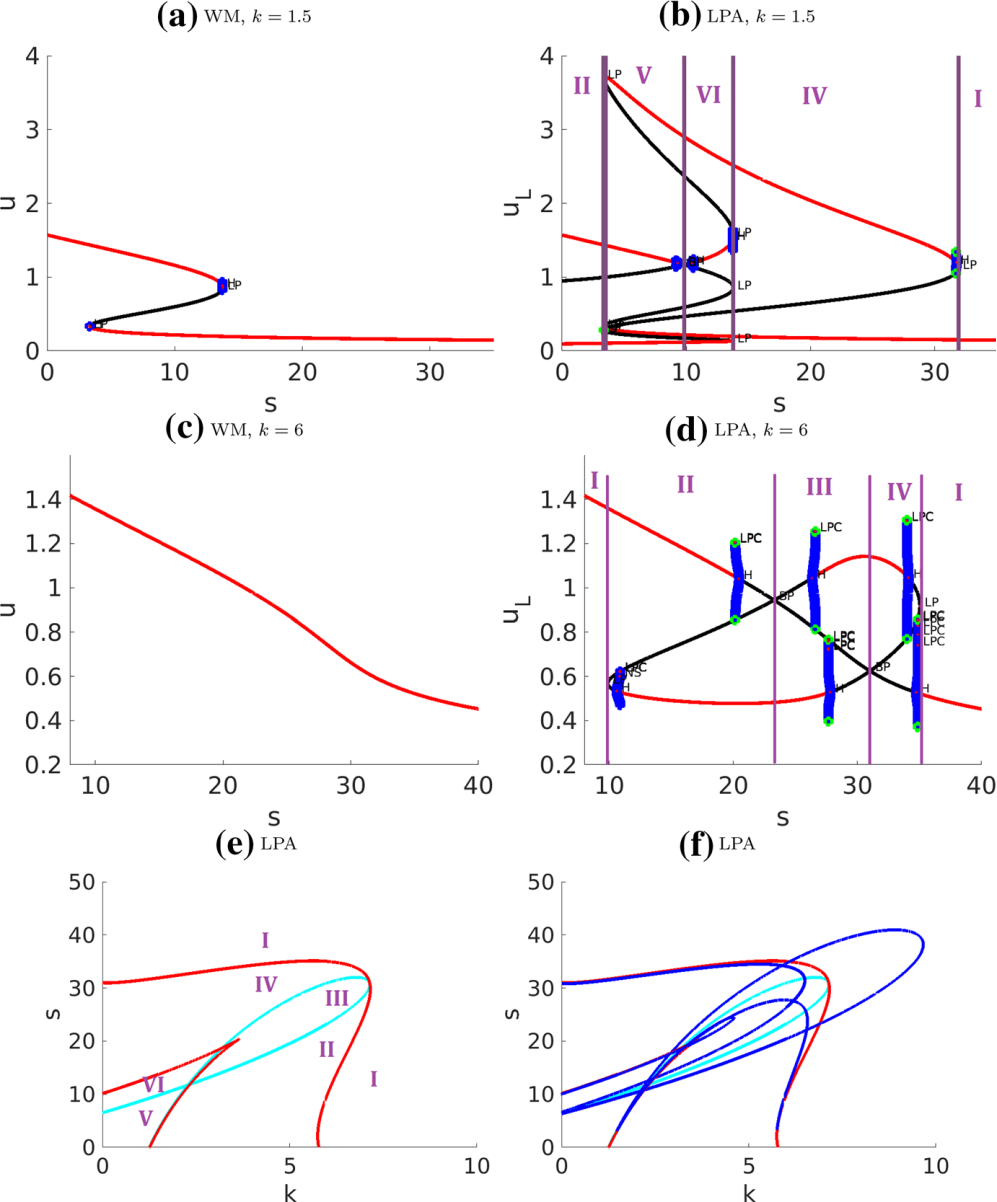
Bifurcation diagrams of the actin feedback (AF) model with respect to parameter *s* **a**–**d** and with respect to *k*, *s* in **e** ,**f**. (In **e**, the Hopf curves are omitted for clarity of the diagram. They are then included in **f**). The narrow regimes are not labelled. The nearly vertical blue curves indicate unstable periodic orbits. The associated simulations are presented in Fig. 10

**Fig. 8:**
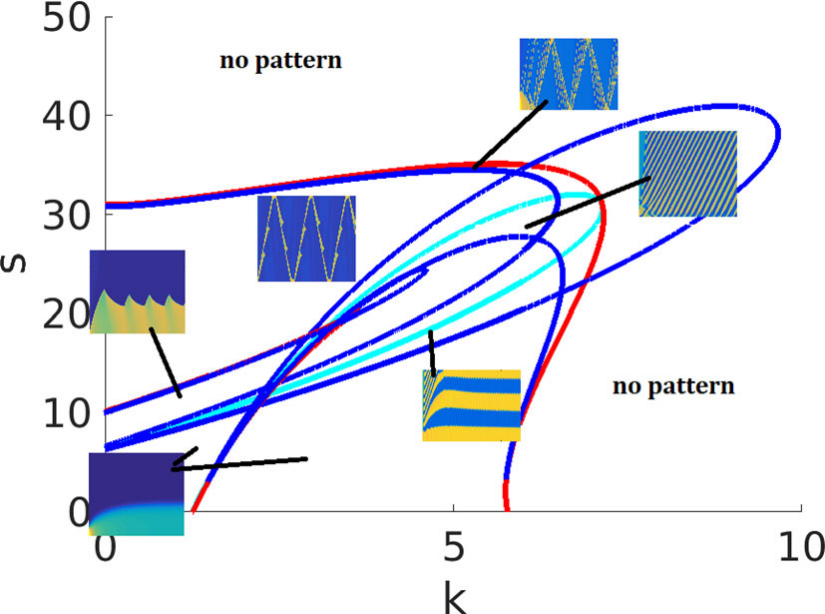
Same as Fig. 7f with the Hopf curves included, and with an indication of patterns in several regimes. A few Hopf curves lie very close to one of the other curves for most of their length, creating some very narrow regimes. The simulation results from Figs. 10 and 11 are identified with their corresponding regions on the parameter plane

The results are shown in Fig. 7 and 8 . We only distinguish between the regimes separated by fold and transcritical curves and omit the Hopf curves, as explained below. We also ignore some very narrow regimes, to concentrate on six major regimes as summarized in Table. 3.

One interesting characteristic of these diagrams is the presence of unstable periodic orbits that emerge as subcritical Hopf bifurcations and exist for very narrow parameter ranges. The unstable cycle enlarges until it collides with a saddle point, turning into a homoclinic orbit to the saddle, and then disappearing. This is known as saddle-loop bifurcation, or homoclinic bifurcation (see (Kuznetsov 2004, Ch.6.2)). Parameter regimes where the periodic solutions exist are very narrow. Hence, while Hopf bifurcations occur, they are unlikely to be playing a major role in the biological application of this model.

We can compare our results to those of Holmes et al. (2012a) (Fig. 5, a LPA diagram in $k-s$ plane containing only one of the Hopf curves). The Hopf curve in Holmes et al. (2012a) corresponds to the dark blue curve on our diagram, which traces the pair of Hopf points on the global branch in Fig. 7d. Furthermore, our diagram (Fig. 8) traces the fold (red) and transcritical (light blue) bifurcation points and hence identifies a larger number of distinct regimes.

Interpreting the LPA diagrams (as in the WP model), we can conclude that a stable local branch in LPA corresponds to a regime of pattern formation in the PDE. Unlike WP, there are multiple possible patterns in this AF model. LPA cannot accurately predict the type of pattern. In particular, the consequence of the subcritical Hopf bifurcations to the full PDE is unclear, possibly suggesting some kind of (quasi-) periodic behavior that we did not fully characterize. In Holmes et al. (2012a); Mata et al. (2013), a parameter scan of the PDE system was included with the LPA diagrams. As previously noted, PDE regimes are not exactly aligned with LPA regimes since $\delta \ne 0$ in the full PDEs.

In summary, in our hands, LPA worked well in identifying no-pattern and WP regimes, but was less useful for predicting the emergence of more complex patterns. Many of those patterns involve interacting waves, which suggests that they are non-linear, non-local phenomena, explaining why LPA cannot account for them.

**Table 3:** Summary of the actin feedback (AF) model regimes identified in Fig. 7

Regime	Classification	Description
I	Stable	One stable GB, no LB
II	Polarizable	One stable GB, one stable LB located above the GB
III	Unstable	The only GB is unstable, two stable LBs located on both sides of the GB
IV	Unstable	The only GB is unstable, one stable LB located above the GB
V	Polarizable	Two stable GBs, three stable LBs: one above both GBs, one in between, and one below both GBs
VI	Polarizable	One stable GB, three stable LBs located on both sides of the GB

For abbreviations see caption of Table 1

### Combined model (CM)

The LPA diagrams for the combined model are very complex, and mostly beyond the scope of interpretation (see Appendix 7.) This is unsurprising given the complex behavior exhibited by the PDE. The bifurcation diagram shown in Fig. 22, which uses parameter values from Table 4 (CM2), contains many limit cycle bifurcations, such as torus and period-doubling. One thing the diagram can provide is the minimum value of *s* required for any non-static patterns (corresponding to the first triplet of Hopf bifurcation in Fig. 22). With *s* below this value, the system behaviour is the same as that of the $s=0$ case, which reduces back to the non-conservative (NC) model.

### Comparison with linear (Turing) stability analysis

Linear stability analysis (LSA) was previously applied by Mori et al. (2008) and Verschueren and Champneys (2017) for the wave pinning and non-conservative models respectively. The relative merits of LSA and LPA have been described in Mata et al. (2013); Holmes (2014) and we briefly summarize some of these in the Appendix.

LPA is only valid in the limit of $\delta \rightarrow 0$. In this limit, LPA contains the Turing stability properties: a branch that is LPA-unstable is also Turing-unstable. See Fig. 23, where we show how the LPA regimes from Fig. 2b line up with Turing regimes. LPA can detect instabilities that require a perturbation of sufficient magnitude (the polarizable regimes), which cannot be detected by Turing analysis. This means LPA can potentially find more types of pattern.

**Table 4:** The parameters in the combined model, their meanings and values for various simulations

Parameter	Meaning	WP	NC	AF	CM2
$\delta$	Diffusion coefficient ratio	0.01			
*L*	Domain length	1	10		
*k*	Basal activation rate	$1.5L^2$		$1L^2-6L^2$	$1L^2$
$\gamma$	Nonlinear activation rate	$30L^2$			
*n*	Hill coefficient	3			2
$\eta$	Inactivation rate	$15L^2$	$5L^2$	$15L^2$	$5.2L^2$
*c*	NC terms on/off	0	1	0	1
$\alpha$	Source strength	$1.5L^2$			
$\theta$	Sink strength	$4.5L^2$			$5.5L^2$
*s*	Actin feedback strength	0		$0-50L^2$	
$\epsilon$	Actin reaction rate	0.1			
$k_n$	Actin activation rate	$24L^2$			
$k_s$	Actin inactivation rate	$7.5L^2$			

*WP wave pinning, NC non-conservative extension, AF actin feedback extension, CM2 one of the parameter sets used for the combined model (CM). All parameters (except L) are scaled to be non-dimensional*

LPA does not predict details of the pattern. We saw this most evidently in the actin feedback (AF) model, where many possible patterns and a large number of parameter regimes exist. Turing analysis predicts pattern initiation, but often fails to specify the final pattern that depends on nonlinear interactions. We give an example of this type for the NC model in Fig. 24. We also indicate how the “minimal patch size” idea from Painter and Hillen (2011) can be used to help predict the final pattern using LSA.

## Numerical simulations

We simulated the model for a static cell in 1D ($0 \le x \le 1$) and 2D ($0 \le x,y \le 1$), and for a motile cell in two spatial dimensions using the Cellular Potts Model (CPM). The four main parameter sets we used for numerical simulations are summarized in Table 4. The selection of values for most of these parameters is based on Holmes et al. (2012a), with $\alpha , \theta$ coming from Verschueren and Champneys (2017), and some modifications guided by LPA and Turing analysis. In contrast to Holmes et al. (2012a) we use a much larger domain size *L*, corresponding to a larger cell and allowing for more complex patterns to develop. We used a shorter domain length *L* to better show the profile of the wave front. With $L=10$ for wave pinning, the pattern appears the same, except the transition is much sharper. The parameters used for the CPM simulations are sometimes slightly different from Table 4. They have been tuned to ensure moderate cell protrusions.

### Simulations in a fixed 1D domain

While 1D simulations for the WP, NC and AF appear in previous works (Mori et al. 2008; Verschueren and Champneys 2017; Holmes et al. 2012a; Mata et al. 2013), we present them here as comparison to the combined model and the 2D case. Results are shown as kymographs, with time on the horizontal axis and space on the vertical axis. Color indicates the levels of *u* and *v* and/or *F* (if $s>0$). For most simulations, we start at a homogeneous steady state (HSS), and perturb the system either with small global noise or with a localized pulse. The first leads to Turing-type patterns, while the latter can lead to the patterns described by LPA.

**Fig. 9:**
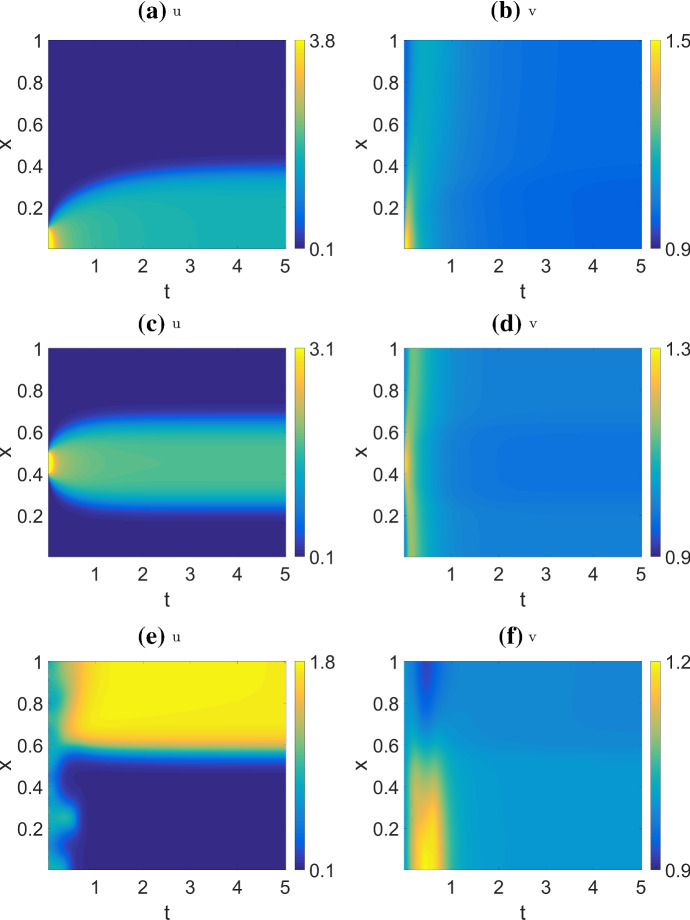
Simulation of the wave pinning model (WP), with parameters from Table 4 (WP). This parameter set corresponds to Regime IIIa as labelled in the bifurcation diagrams Fig. 4b and 5, which is classified as a polarizable regime. The patterns produced in the other polarizable regimes are qualitatively similar. Initial condition: $v=1$, $u=0.102$ with perturbation $u=6$ for (**a** ,**b**) $0 \le x \le 0.1$; (**c** ,**d**) $0.4 \le x \le 0.5$; (**e** ,**f**) random noise, $u=0.834\cdot \epsilon (x)$. Note that not all initial conditions result in wave pinning: a small perturbation from the HSS will simply decay and no pattern forms. The behaviors shown in **a**, **b**, **e**,**f** correspond to solutions shown in Fig. 2 of Mori et al. (2008)

Figure 9 shows the results for the WP model. Observe that for the first two cases (a,c), the initial perturbation decays considerably, but nevertheless this results in formation of a pattern associated with polarization. The random initial condition (e) also results in a polarized steady state. In these simulations, *u* can vary greatly across the domain while *v* becomes nearly uniform, as expected given its much faster rate of diffusion.

**Fig. 10:**
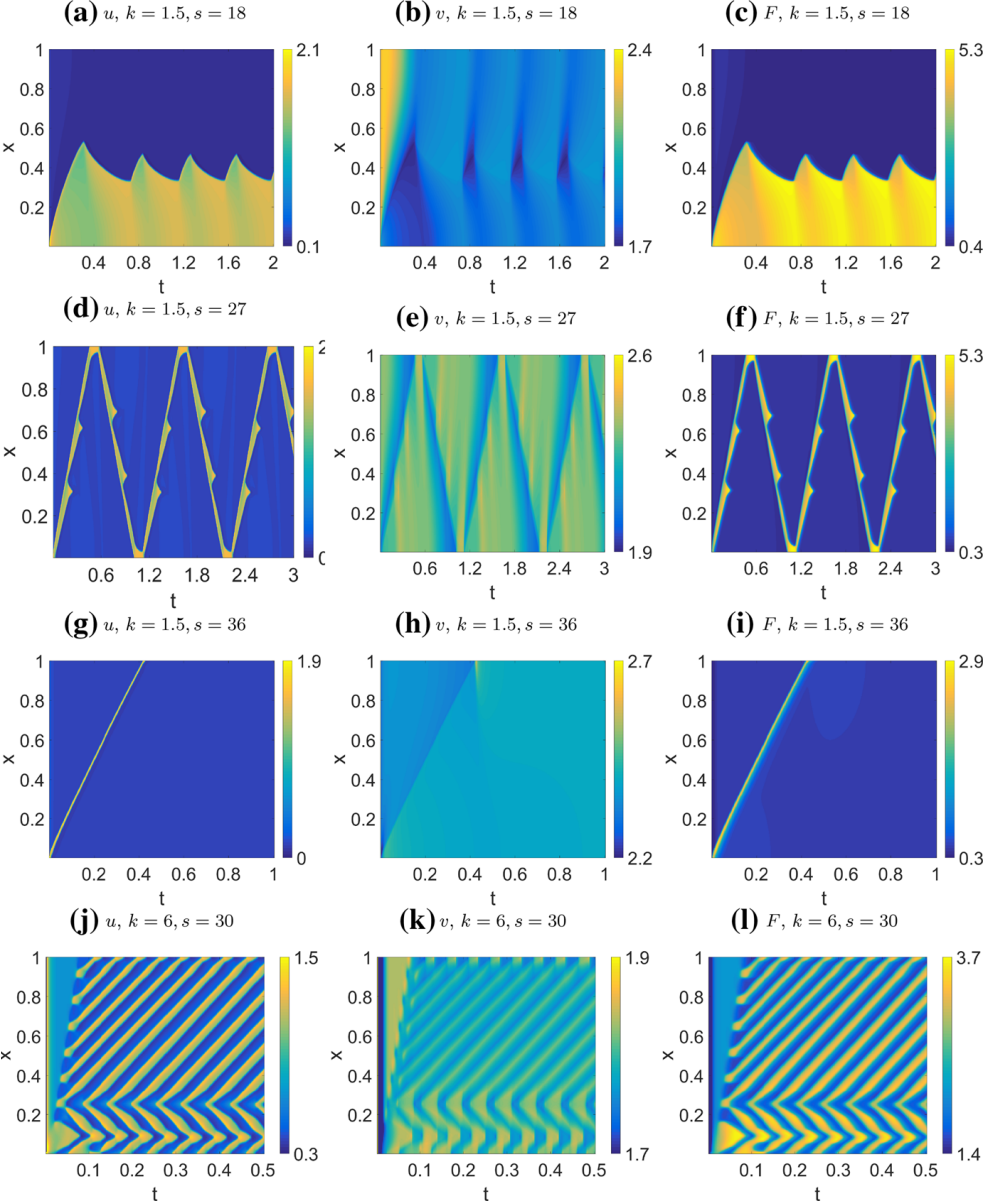
Simulations of the actin feedback model (AF) with parameters from Table 4 (AF) (*s*, *k* as indicated on labels), and default initial conditions. Each row corresponds to one parameters set, showing *u*, *v*, *F* (left to right). We also indicate the regimes each parameter set corresponds to in bifurcation diagram Figs. 7 and 8 . We observe four behaviors by varying *k* and *s*: **a**–**c** Wave pinning with oscillating front (WPO), within Regime IV but near the boundary with Regime VI; **d**–**f** Reflecting waves (RW), Regime IV; **g**–**i** Single pulse absorbed at boundary (SP), within Regime I but near the boundary of Regime IV; **j**–**l** Persistent wave trains (WT), Regime III. We used a larger domain length than Holmes et al. (2012a), leading to a richer set of patterns

Figure 10 shows the results for the actin feedback (AF) model. The default initial conditions are $u=0$ except $u=4$ for $0 \le x \le 0.01$, $v=2.5$, $F=0$. We are not initializing near a stable HSS because doing so usually does not result in patterning. The patterns observed are quite sensitive to initial conditions. In addition to simple wave pinning observed at low *s* (not shown), the system displays four qualitatively different behaviors: (1) wave pinning with oscillating boundary (WPO), where polarization occurs as in wave pinning, but with an oscillating front position; (2) reflecting pulse (RW), where a single pulse traverses the domain at constant velocity and gets reflected back at the boundary; (3) a single pulse (SP) that is absorbed at a boundary, before the system returns to HSS; (4) a wave train (WT), that originates either at a boundary or in the interior of the domain, propagates with constant velocity and gets absorbed at a boundary.

In general, the spatial profile of *F* lags behind *u*, as expected, since it is a slow variable depending on *u*. The pattern in *v* is usually opposite that of *u*, i.e, *v* is high where *u* is low, and vice versa. Moreover, the gradient of *v* tends to be much shallower than *u* due to the faster diffusion of *v*.

**Fig. 11:**
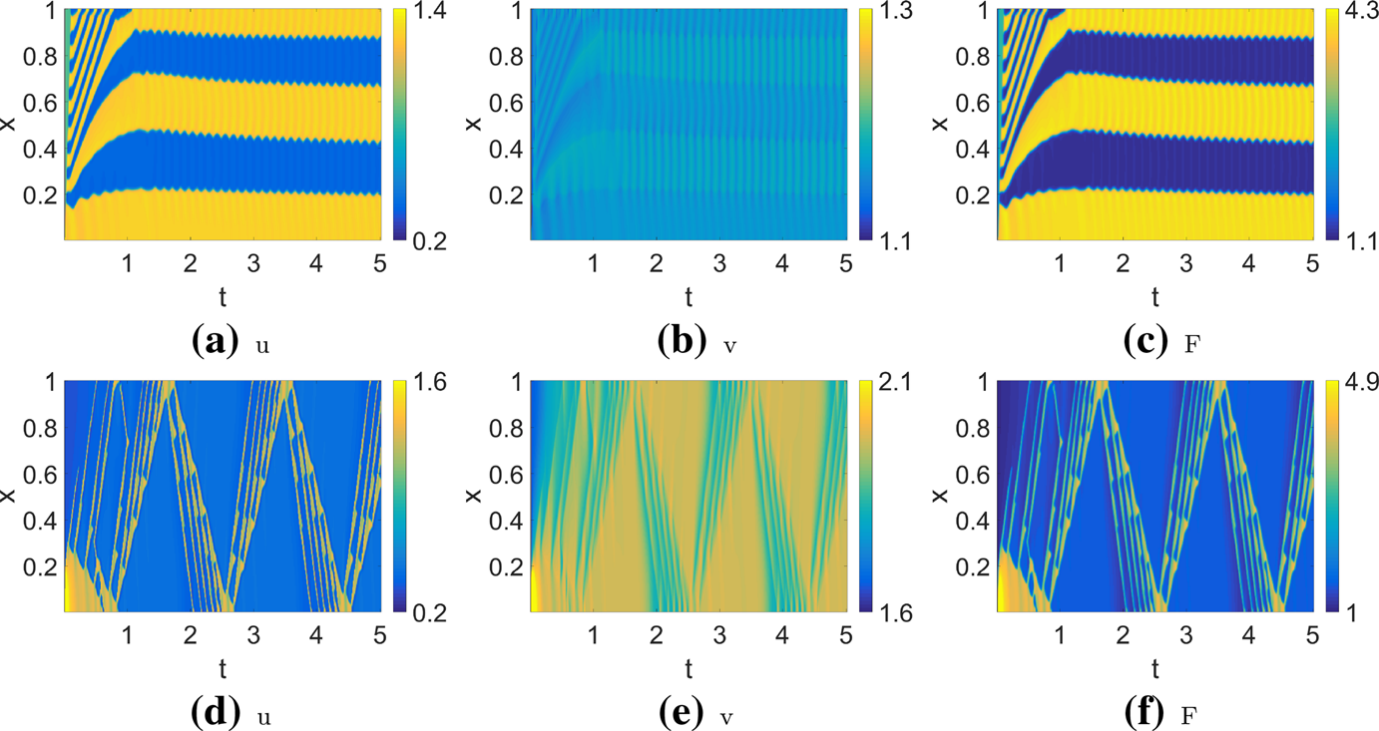
Exotic patterns observed in the actin feedback (AF) model. Parameters as in Table 4 (AF) but varying *k*, *s*. Both of these are located near regime boundaries in the bifurcation diagram Fig. 8. **a**–**c** $k=5, s=10$, default initial conditions. The pattern resembles WPO but with several subregions; **d**–**f** $k=5, s=30$, default initial conditions with excitation region $0\le x \le 0.1$. The resulting pattern is similar to RW but with a group of four pulses traversing the domain

Some other more complex patterns are shown in Fig. 11. These share some characteristics with the simpler patterns. The patterns shown in (a–c) are similar to (WPO), but the domain is divided into five regions instead of two, with an initial transient reminiscent of (WT). The patterns in (d–f) can be seen as a group of four reflecting pulses (similar to RW) rather than one. Compared to Holmes et al. (2012a), we find a richer range of patterns using a similar parameter set (with different scaling). The main difference is that the larger domain used here, $L=10$, allows more space for pattern to develop. (In (Holmes et al. 2012a), $L=1$, so patterns are more confined and boundary effects are prominent.)

**Fig. 12:**
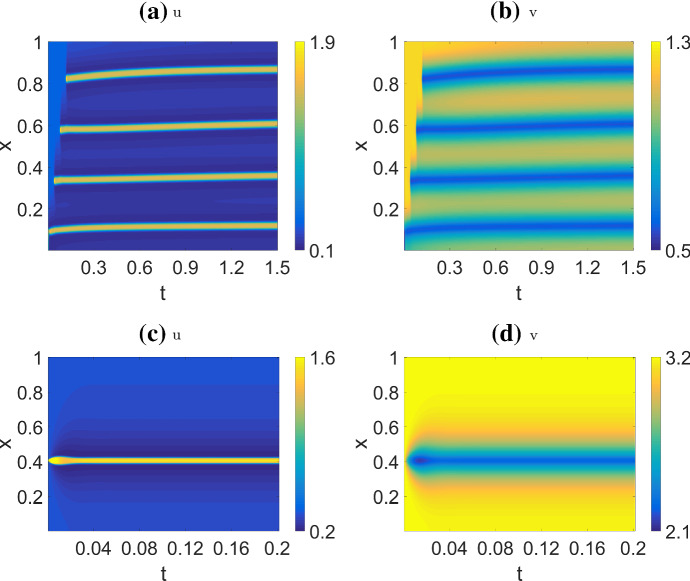
Simulation of the non-conservative model (NC) with **a**, **b** default parameters (Table 4 (NC)), corresponding to the unstable Regime III as identified in the bifurcation diagram Fig. 6; **c**, **d** $\gamma =15L^2, \eta =15L^2$, which corresponds to the polarizable Regime II. Initial condition: **a**, **b** $u=u_*$ except $u=1$ on $0 \le x \le 0.1$, $v=v_*$. **c**, **d** $u=u_*=0.33333$ except $u=10u_*$ on $0.4\le x \le 0.41$, $v=v_*=3.19298$. In **a**, **b**, the formation of a peak on the left triggers some new peaks farther away, until space runs out. Once all peaks form, they shift slightly to be evenly spaced. In **c**, **d**, the single initial peak persists, without triggering new peaks. We refer to this as the soliton solution

**Fig. 13:**
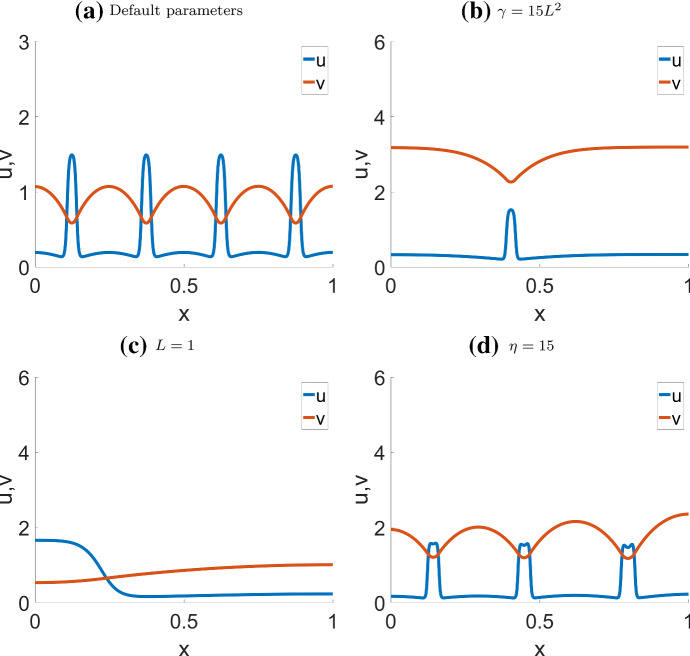
Final steady state pattern of the non-conservative model (NC) with most parameters from Table 4 (NC), except the parameters indicated on the labels. **a** and b correspond to the steady state of Fig. 12**a**, **b** and c, d respectively. In **c** the shortened domain results in wave pinning; **d** Higher inactivation rate $\eta =15$ results in bifurcating peaks. **a**, **b** corresponds to Fig. 5a, d of Verschueren and Champneys (2017), respectively

Fig. 12 shows two typical patterns in the NC model: a static, Turing-type pattern consisting of a series of evenly spaced spikes, and a single spike “soliton” pattern. The final profiles of these two patterns are shown in Fig. 13a,b. The domain length, *L*, must be large enough to support such patterns. If *L* is too small to support a full period of the pattern, the result would be simple polarization similar to wave pinning (Fig. 13c). Using a higher rate of inactivation $\eta$, or a smaller diffusion ratio $\delta$ can result in spikes that split into two, as shown in Fig. 13d.

**Fig. 14:**
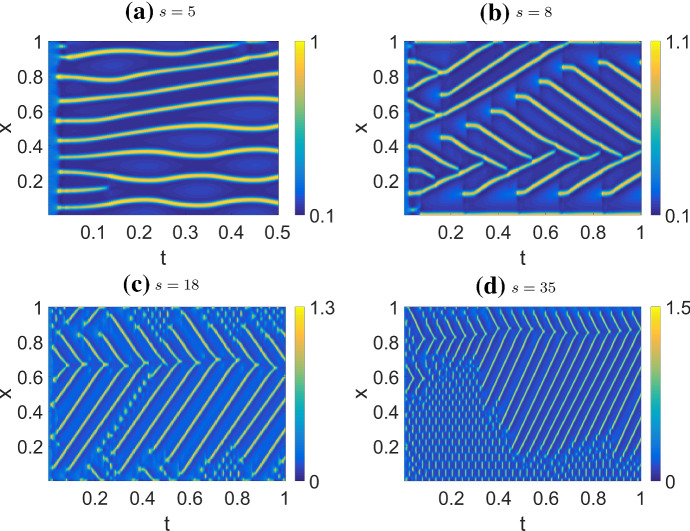
Simulations of the combined model (CM), where only the profiles of *u* are shown. The profiles of *v* show mostly similar patterns. Parameters as in Table 4 (CM2) but varying *s*, and HSS+noise initial condition as described in the text. As we increase the actin feedback strength *s*, the behavior transitions from slowly moving, repelling peaks to colliding peaks. At higher *s*, there is a rapidly oscillating standing wave pattern in some parts of the domain

For the combined model, we use parameters from Table 4 (CM2), mostly similar to the NC case. In Fig. 14, we show the effect of increasing *s* (strength of actin feedback) on system behavior. With *s* low enough, the system behavior resembles the $s=0$ case of a static, spatially periodic pattern, as in the NC case. For increasing *s*, the peaks begin to move with constant velocity by themselves, repelling one another when too close. For moderate values of *s*, the peak repulsion is strong enough that peaks reverse their direction of motion if on a collision course (Fig. 14a). At higher *s*, they collide (Fig. 14b). At even higher *s*, we observe a localized standing wave pattern that oscillates rapidly in Fig. 14c, and even more prominently in (d).

In sumary, CM behavior is close to AF behavior only when *s* is large, as in Fig. 14 c,d. This is unsurprising since *s* controls the magnitude of actin feedback. When *s* is small (Fig. 14 a,b), the effect from the nonconservative term dominates and we can observe the same periodic spikes characteristic of the NC model. The primary effect of increasing *s* is to allow the spikes to move with increasing speed, and this seems to be true in general, as we observed under a variety of parameter sets.

### Simulations in a fixed 2D rectangular domain

**Fig. 15:**
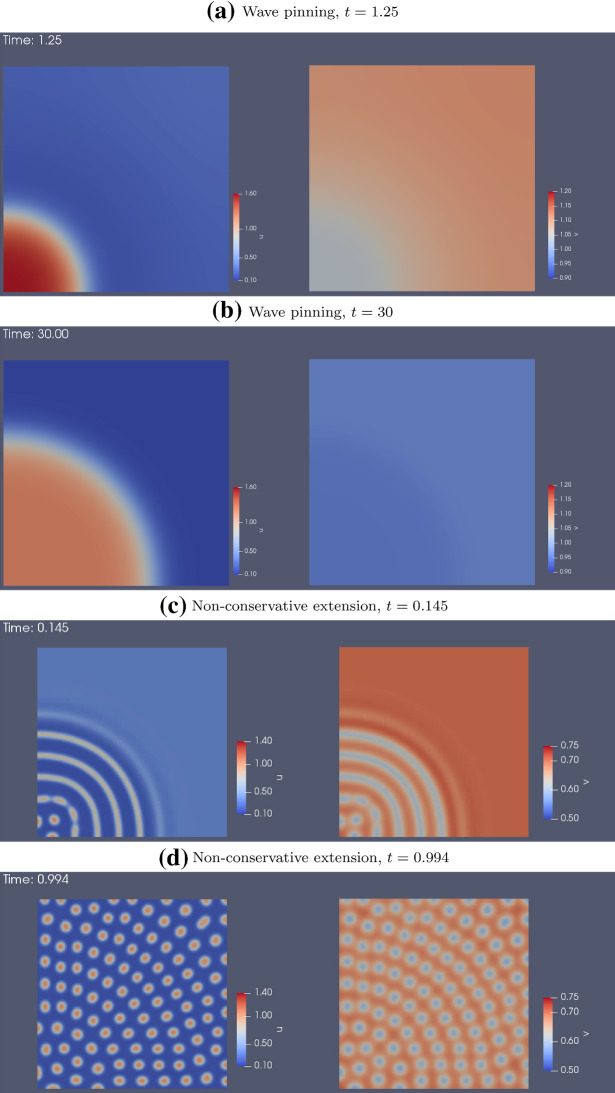
Simulations of the wave pinning **a**, **b** and non-conservative **c**, **d** models in a 2D static square domain, using the same parameters as in 1D (Table 4 (WP) and (NC)). Left: *u* , Right: *v*. For each model, two snap shots are shown: one when the pattern begin to take shape, and another after the system reached steady state

In two spatial dimensions, we use the same parameters as in 1D. For the WP model, we start at HSS and perturb one corner of the domain. The pattern we observe (Fig. 15a, b) is a direct analogue to the 1D case (compare to Fig. 9a, b): *u* initially spreads out from the corner as a 2D travelling wave, and that is eventually pinned along a front determined by the initial conditions.

We use a similar initial condition for the NC model. Based on 1D simulations, we expect evenly-spaced stripes to form around the corner as concentric rings, as happens initially (Fig. 15c). However, these rings quickly break up into spots (Fig. 15d). The spots spread out, and then settle into a steady state. We have not found any parameter sets for stable ring patterns. The patterns are insensitive to the shape of the domain. Simulations on circular, rectangular and other domains with simple shapes produced patterns with the same qualitative characteristics (not shown).

For CM, we initialize the system at HSS and perturb with noise. Figure 17 shows the simulation results. With a low *s*, the pattern is indistinguishable from the static spots under the non-conservative model. As *s* increases, the spots become mobile and repel each other as in the 1D case. In 2D, as *s* is increased further, the spots transitions to spiral waves.

**Fig. 16:**
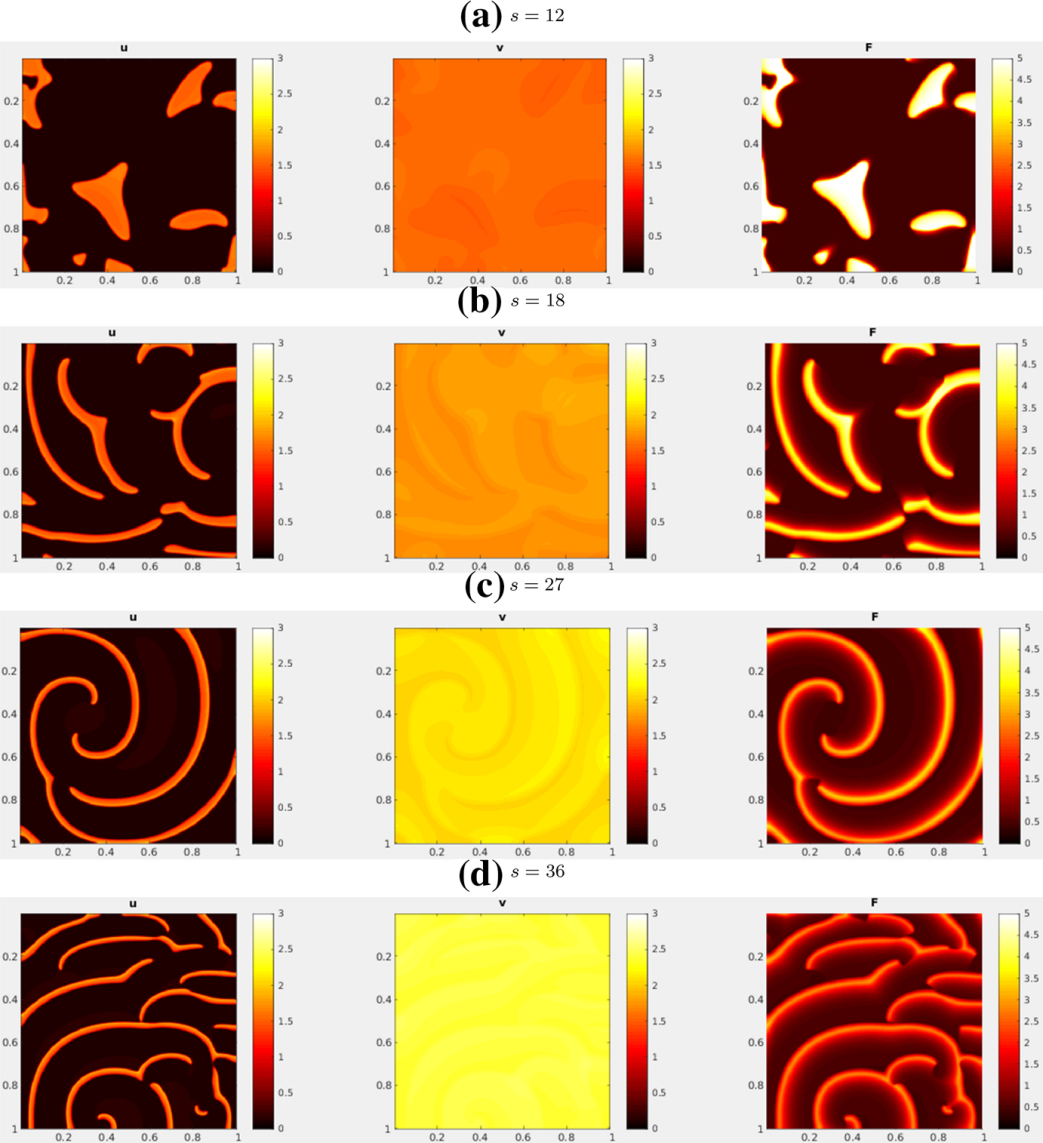
2D simulations of the actin feedback (AF) model, with parameters from Table 4 (AF) and initial conditions described in the text. These snapshots are taken after the patterns have fully developed. As *s* increases, blobs transitions into thinner and thinner spiral waves. See movies at https://imgur.com/a/61GwiA9

We also arrived at the AF model by initializing the CM model at HSS plus global noise and $c=1$. After a pattern starts to form, we gradually decreased *c* to 0 to arrive at the AF model. (In our hands, this produced more robust results, with patterns that persisted). Figure 16 shows a few snapshot of the simulations. For low *s*, the pattern resembles slowly drifting and deforming blobs. As *s* increases, the pattern transitions into spiral waves with decreasing width.

**Fig. 17:**
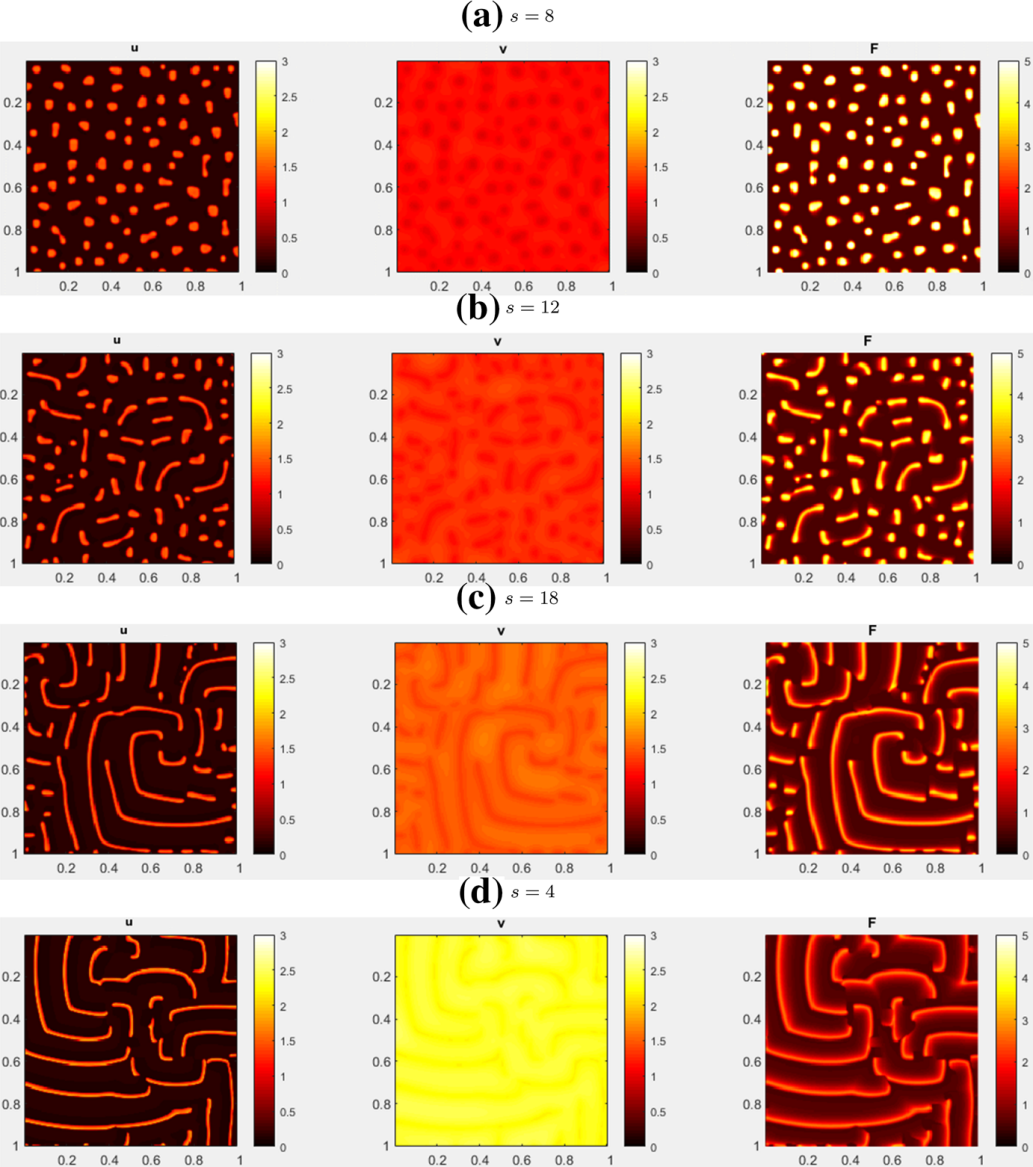
Simulations of the combined model (CM) in 2D, with parameters from Table 4 (CM2) and HSS + noise initial condition. There is a transition from spots to spiral waves near $s=12$. See movies at https://imgur.com/a/a0u57GQ

### Simulations in a 2D deforming domain

As a final set of numerical experiments, we simulate the revised WP models in an evolving 2D domain. The boundaries deform in response to the chemical levels close to the boundary. We use the Cellular Potts Model (CPM) for these examples.

The CPM is a common method for tracking an evolving shape such as morphology of a motile biological cell (Scianna et al. 2013). (In 2D, the cell is “viewed from above” as it migrates on a flat 2D surface.) The neighbourhood of each point inside the shape represents a 2D projection of some small cylinder in 3D, containing both membrane and cytosol. Hence, active and inactive GTPases (*u* and *v*) coexist at every point inside the given shape, as they do in our fixed domain 2D simulations.)

Commonly, for the CPM, a scalar Hamiltonian, analogous to a potential is assumed to depend on the area and perimeter of the cell, as well as the interface contact with other cells or empty space. Changes to the boundary of the cell are accepted or rejected stochastically, according to the net changes in the Hamiltonian, as described in the Appendix. Our simulations include the following additional features: (1) solving the reaction-diffusion PDEs inside the evolving domain with Neumann boundary conditions at the cell boundaries and (2) modifying the Hamiltonian to depend on the local RD variables.

In real cells, actin polymerizes into F-actin, and promotes protrusion of a cell edge. Hence, we link the F-actin variable *F* in the model to forces on the cell boundary, (by superimposing a chemically-dependent potential $H_0=\pm \beta F$ for retractions(+) vs extensions(-) on the basic Hamiltonian, see Appendix). In variants of the model that do not explicitly track F-actin, we assume that the GTPase *u* plays a similar role (i.e., that *u*, like the GTPase Rac, locally promotes cytoskeleton assembly, creating a protrusive force at the cell edge).

**Fig. 18:**
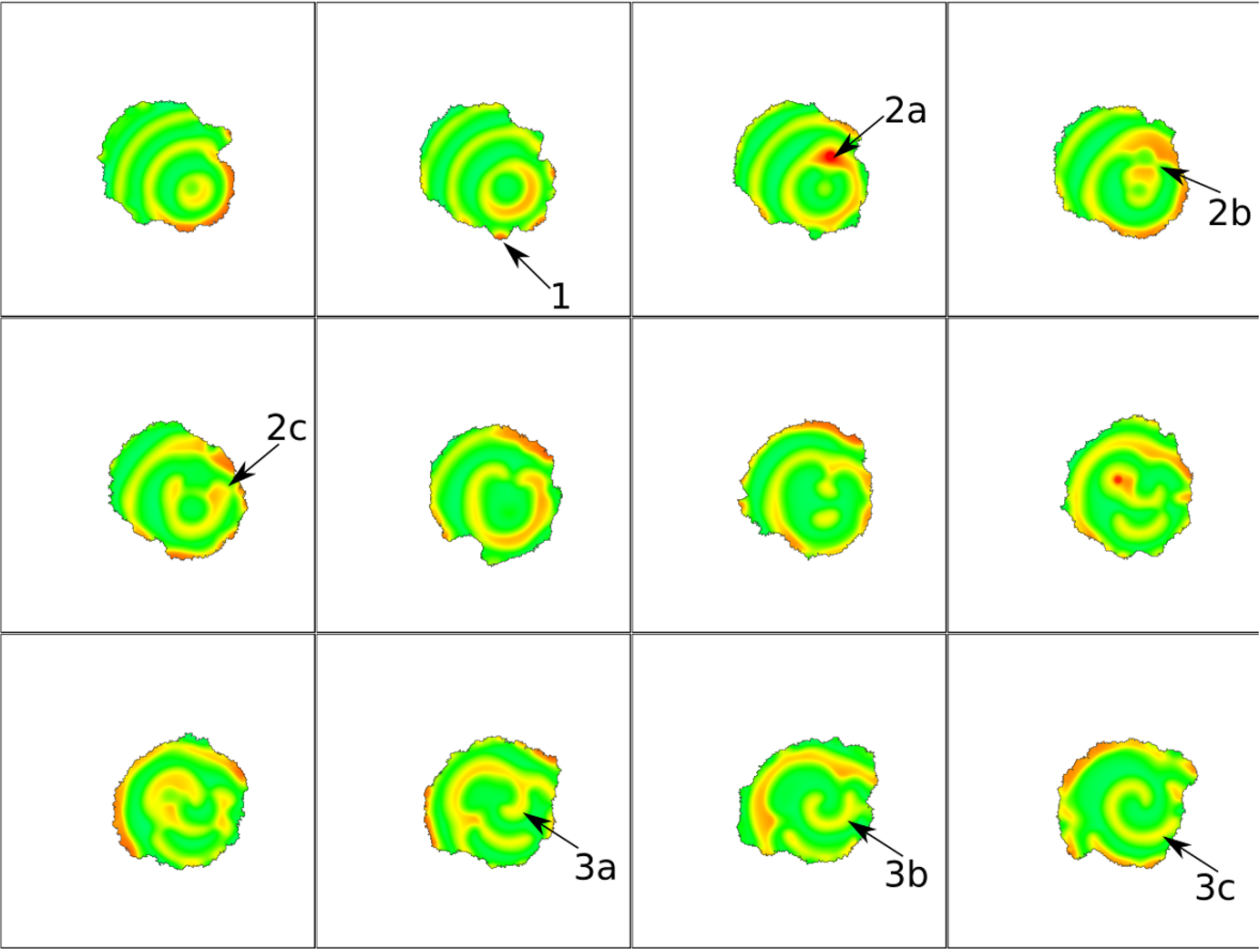
Snapshots of 2D CPM simulation with parameters from the absorbing waves in a static domain (AF model). Visualized is F-actin (*F*) that promotes protrusions ($H_0=\pm \beta F$). Arrows indicate examples of interesting dynamics: (1) F-actin wave pushes membrane, (2)(**a**–**c**) A spot (2**a**) breaks into two waves (2**b**) and one wave changes direction (2**c**), (3**a**–**c**) a spiral starts to form. Snapshots are 20 MCS apart. Parameters are: $\delta = 0.06$, $L=1$, $k=6$, $s=30$, and the rest from Table 4 (AF). CPM Parameters are: $a=12000$, $\lambda _a=2$, $p=500$, $\lambda _p=20$, $J=50$, $r=3,\xi (r)=18$, $\beta =150$, $T=100$. Movie link https://imgur.com/a/7OmgctR

Simulations are initiated with a circular cell and internal variables close to HSS. Other initial conditions produce similar dynamics. We first considered a parameter regime that produced the absorbing wave simulations in the static domain. Figure 18 (left to right, top to bottom) shows a time series of the corresponding CPM simulation with the same parameters. A peak of active GTPase, *u*, initiated at a random location produces a series of circular ripple waves. Waves of F-actin or of *u* (here associated with the GTPase Rac) that impinge on the cell edge (1) lead to local protrusion. A new burst produces additional waves that split and move towards the cell edge (2a,b,c). Colliding waves sometimes create local spiral waves (3a,b,c) in the cell interior, and lead to continued cell shape deformations. A movie of the same sequence can be found at https://imgur.com/a/7OmgctR.

**Fig. 19:**
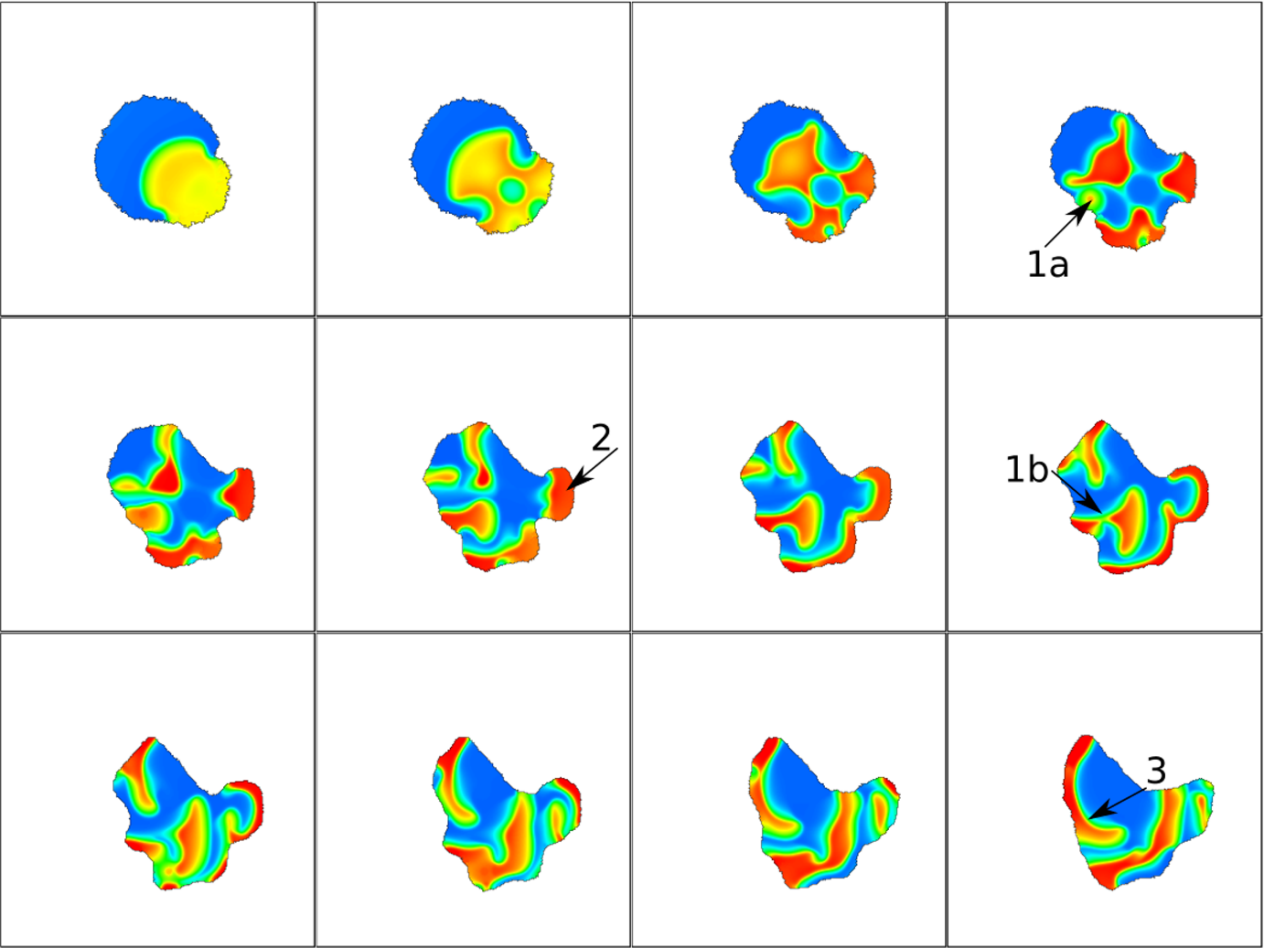
Snapshots of 2D CPM simulation with parameters from the oscillating waves in a static domain (AF model). Visualized is F-actin (*F*) that promotes protrusions ($H_0=\pm \beta F$). Arrows indicate examples of interesting dynamics. (1)(**a** and **b**), a spot extends towards to membrane **a** and later breaks into two and one wave moves inwards **b**, (2) a wave creates a big protrusion, (3) a wave hits the cell edge and spirals in. Snapshots are 20 MCS apart. Parameters are as in Fig. 18, but with $k=1.5$, $s=18$. CPM parameters are as in Fig. 18, but with $\beta =50$. Movie link https://imgur.com/a/eIAjr59

A new random burst appears (Arrow 2a) and produces waves in two directions (Arrow 2b). When waves collide, they break, amplify, move left and right (Arrow 2c) and eventually give rise to a spiral wave. Because the resulting spiral wave has a lower magnitude, there is weaker effect on the boundary at this time. In Fig. 19, we show a time series for parameters that produced oscillating waves in the static domain. As before, the initial burst is in the lower right, and a new burst (Arrow 1a) breaks apart into two waves that broaden. We find a protrusion that is much broader than in Fig. 18 (Arrow 2). Wave absorption is lower than in Fig. 18, so the cell edge is pushed further out.

**Fig. 20:**
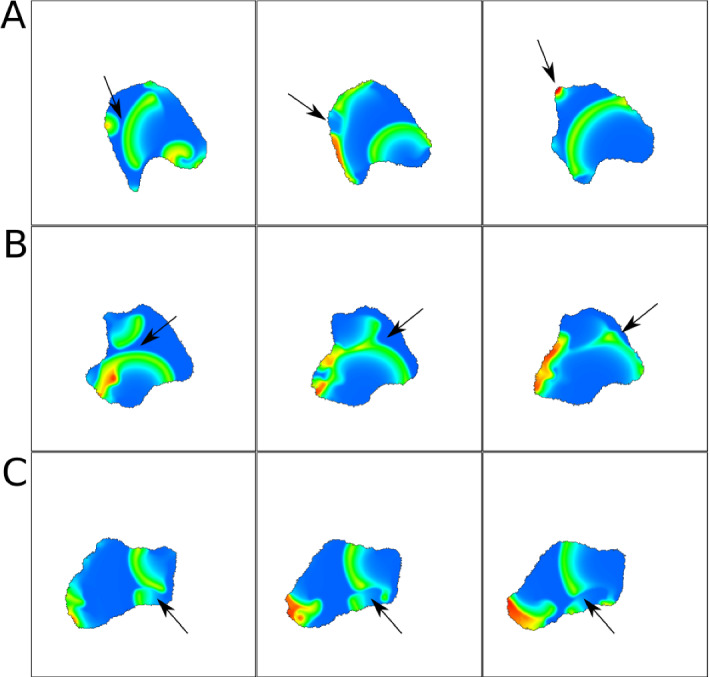
Snapshots of 2D CPM simulation with parameters from the reflecting waves in a static domain (AF model). Visualized is F-actin (*F*) that promotes protrusions ($H_0=\pm \beta F$). Snapshots in A,B,C are 10,20,20 MCS apart respectively. Arrows indicate examples of interesting dynamics. **a** a wave hits a spot and breaks into two, **b** Two waves merge and change direction, **c** Part of a wave disappears as it encounters a spot. Parameters are as in Fig. 18, but with $k=1.5$, $s=27$. CPM parameters are as in Fig. 18, but with $\beta =50$. Movie link https://imgur.com/a/FDCn3NY

Figure 20 shows results for parameters corresponding to reflecting waves in a static domain. Here, because the cell boundary moves outwards, the waves are usually absorbed, rather than reflected. Occasionally, if the wave hits the cell edge tangentially, it is reflected (e.g. at 19 sec in the movie, upper left corner). We furthermore observe three new wave dynamics in a moving cell with random bursts. A wave can break apart when it hits a burst (A), waves can merge (B), or avoid each other (C).

**Fig. 21:**
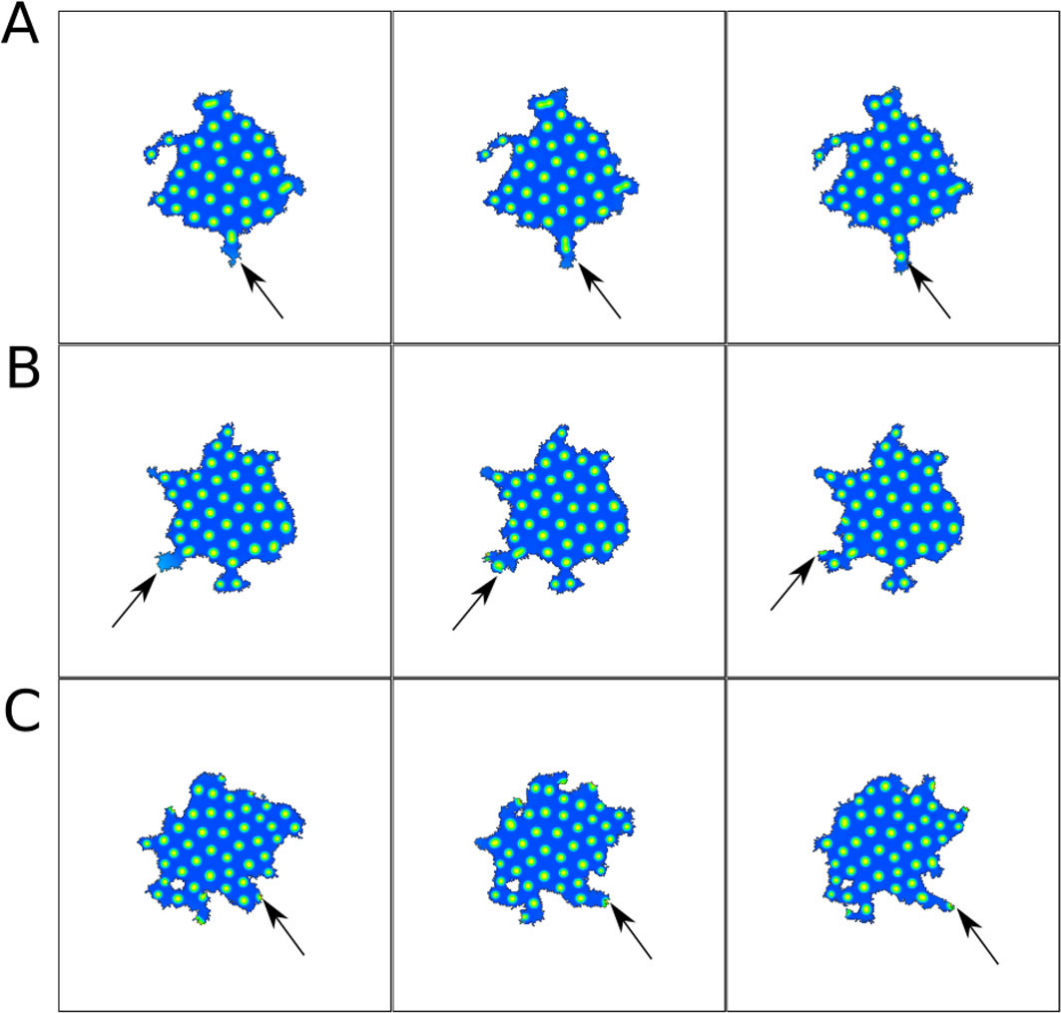
Snapshots of 2D CPM simulation of the NC model. Visualized is Rac (*u*) that promotes protrusions ($H_0=\pm \beta u$). Snapshots in A,B,C are 10,5,25 MCS apart respectively. Arrows indicate examples of interesting dynamics. **a** a spot breaks into two, **b** a new spot is created in a protrusion, **c** a spot leads a protrusion to form. Parameters are: $\delta = 0.1$, $\eta =60$, $k=6$, $\gamma =120$, $\theta =18$, $\alpha =6$, and the rest from Table 4 (NC). CPM Parameters are: $a=10000$, $\lambda _a=0.02$, $p=1000$, $\lambda _p=0.04$, $J=40$, $r=3,\xi (r)=18$, $\beta =200$, $T=20$. Movie link https://imgur.com/yUUEgQD

As a last experiment, we simulate the formation of spots in the NC model (Fig. 21). Since this model has no F-actin variable, we base the edge protrusion on the active GTPase *u* (assumed to act like Rac in promoting local cytoskeleton assembly). The spots are highly dynamic and, as expected, lead to the formation of small protrusions (“filopodia”) (C). Furthermore, edge deformation also causes the spot pattern to change. When a protrusion forms (stochastically or by locally elevated *u*), the spot in a region close to the protrusion can split into two, one of which moves into the protrusion (A). We also see formation of new spots inside protrusions (B).

## Discussion

In summary we have explored extensions of the wave-pinning model (WP) (Mori et al. 2008, 2011) that coupled the non-conservative variant proposed by Verschueren and Champneys (2017) (NC) and the actin feedback (AF) model of Holmes et al. (2012b). We found that the combined model (CM) borrows features from both, with moving peaks and wave trains, as well as more complex hybrid dynamics. At the same time, we were unable to find blinking localized spots as observed experimentally in (Robin et al. 2016). Despite the fact that the work of Robin et al. (2016) points to interactions of F-actin with the GTPase Rho, other unknown factors, missing in our model, should be considered to explain such behavior.

We used the local perturbation analysis (LPA) on each model variant. As noted before (Holmes et al. 2015), LPA recovers Turing analysis. States that are LPA stable are also Turing stable. LPA helps to identify potentially interesting parameter ranges, including polarizable regimes that cannot be detected by Turing analysis. At the same time, LPA does not predict details of patterns that emerge, nor accurate bifurcation points in the full PDE system. LPA achieved varying degrees of success for all models we tested. For the nonconservative model, LPA was able to distinguish the stable, polarizable and Turing-unstable regimes. The only caveat is that the Hopf bifurcation present in the LPA system does not necessarily correspond to a bifurcation in the PDE system. For the AF model, LPA could identify several regimes corresponding to distinct behaviors (Fig. 8). The complexity of the LPA ODE system and the associated bifurcation diagrams meant that we could not “read off” PDE behavior from the resulting LPA bifurcation diagrams. For CM the diagram is simply too complex to decipher. Although we always rely on numerical simulation to match behaviors to regimes, LPA still proves to be a handy shortcut to avoid exhaustive parameter sweeps, and to help locate the interesting regimes in the PDEs.

We also encountered examples where LPA identified apparent bifurcations that did not materialize as true regimes of behavior in the PDEs.

As a second innovation, we simulated all model variants in 2D on both a static and a deforming domains, and summarized the observed patterns in Table 5. Previous work (Verschueren and Champneys 2017; Holmes et al. 2012a) was concerned with fixed 1D domains for the PDEs. We hence showed that the patterns for the nonconservative (NC) model were primarily spots, not bands, whereas the AF model, while appearing to be less robust, produced a variety of moving peaks, bands, and waves, including spiral waves. All these patterns differ significantly from the simple pinned wave of the original model in Fig. 2

Finally, our simulations of the models in deforming domains mimicking a motile cell allowed us to consider the connection to experimentally observed cell polarization (Jilkine and Edelstein-Keshet 2011; Rappel and Edelstein-Keshet 2017) and waves of actin (Inagaki and Katsuno 2017). We showed that the internal dynamics of the models (and in particular the actin feedback model) have an interesting consequence on the motility of a “model cell”. Indeed, the waves of high and low signaling levels led to formation of cellular protrusions, and gave rise to nontrivial motion in the deforming cell.

## Figures and Tables

**Fig. 22:**
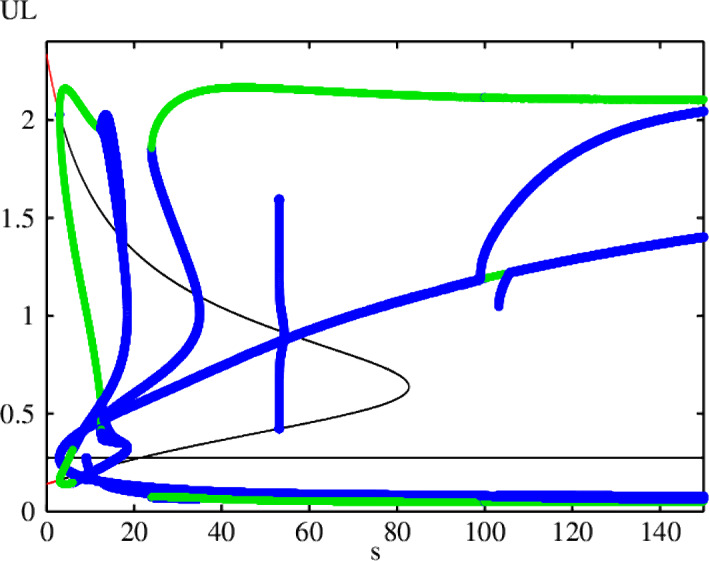
LPA bifurcation diagram for the combined model (CM) with respect to the parameter *s*. Other parameters as in Table 4 (CM2). There are many apparent branches of periodic solutions. In a parameter range around $s=20$, there are no stable equilibria nor stable periodic solutions even though the system remains bounded, which suggests the presence of chaos

**Fig. 23:**
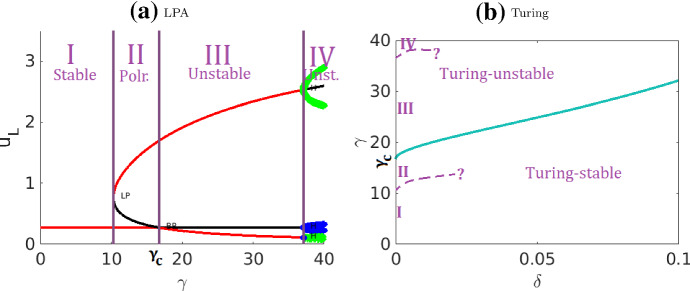
Comparison of LPA and Turing bifurcation diagrams for the non-conservative model. **a** is a zoom of the LPA diagram from Fig. 6b. **b** is the Turing bifurcation diagram reproduced from Fig. 5 of Verschueren and Champneys (2017), using the same parameters. Observe that both the LPA-stable (I) and the LPA-polarizable (II) regimes in **a** located to the left of $\gamma _c=16.765$ correspond to the Turing-stable regime below the blue curve in (**b**). The LPA-unstable regimes (III, IV) correspond to the Turing-unstable regime above the curve. The curve passes through $\delta = 0, \gamma =\gamma _c$. The bifurcation boundary between Regimes I and II, and between III and IV cannot be detected by Turing analysis. Given that numerical simulations have shown that the PDE produces the same behavior (Fig. 13a) in both Regimes III and IV, it is possible that these are not distinct regimes for the PDE. Overall, the LPA diagram (a) can be seen as a vertical slice of the Turing diagram **b** at $\delta =0$, with additional bifurcation boundaries that separates the LPA-stable and LPA-polarizable regimes

**Fig. 24:**
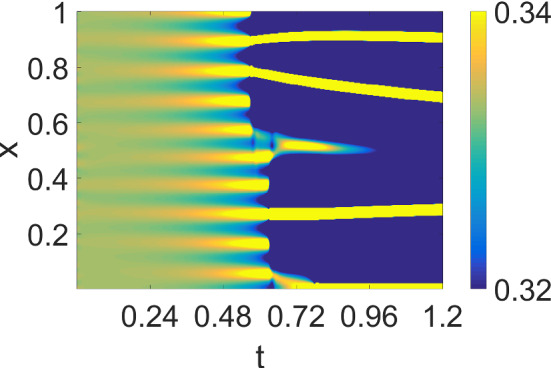
Simulations for the non-conservative model with random initial conditions $u=u_* (1+\text{ Unif }(-0.01,+0.01)), v=v_*$ with default parameters from Table 4 (NC) except $\gamma =25$. The color range is chosen so that the precursor pattern is more visible. The rapid transition from the shallower, higher frequency precursor pattern to the final pattern can be clearly seen

## Appendix: LPA diagram for the NC model

The LPA diagram for the nonconservative model (NC) is given in Fig. 22. Due to the many intertwined bifurcations, it is difficult to interpret this diagram, and we present it here only to demonstrate the limitations of LPA.

**Table 5:** Summary of observed patterns in static and deforming domains. The “type of pattern” applies primarily to patterns observed in static simulations, and are not exactly the same as the corresponding deforming domain simulations using similar parameters

Types of pattern	Model	Static domain figures	Corresponding deforming domain figures
Wave pinning	WP	2, 9, 15a, b	3
Wave pinning with oscillating front	AF (1D)	10a–c	19
Reflecting pulse	AF (1D)	10d–f	20
Wave train	AF (1D)	10j–l	18
Spiral waves	AF, CM (2D)	16, 17	18, 19, 20
Stationary,periodic spikes(1D)/Spots(2D)	NC	12a,b, 15c, d	21
Soliton (single static spike)	NC (1D)	12c,d	–
Moving spikes(1D)/spots(2D)	CM	14, 17	–

## Appendix: Relation between Turing and LPA

Linear stability analysis (i.e. Turing analysis) (Turing 1952) is a more traditional method of analyzing the stability of reaction-diffusion systems which focuses on the stability of a HSS in response to perturbations in the form of a global noise with infinitesimal height.

Here we examine the relation between Turing and LPA. This was previously done by Mata et al. (2013) for the two-variable case in terms of eigenvalues, and for the general case by Theorem 4.1 of Holmes (2014). (The proof for this theorem is quite involved.) Here we present a much more elementary argument for the two-variable case.

Suppose in a general reaction-diffusion PDE with a slow-diffusing quantity *u* and a fast-diffusing quantity *v*. Assume that time has been rescaled so that the diffusion coefficient of the fast quantity is 1.

$$\begin{aligned} \frac{\partial u}{\partial t}&= \delta \nabla ^2 u +f(u,v),\\ \frac{\partial v}{\partial t}&= \nabla ^2 v +g(u,v) . \end{aligned}$$

The corresponding well-mixed (WM) system is

$$\begin{aligned} \frac{\partial u}{\partial t}&= f(u,v),\\ \frac{\partial v}{\partial t}&= g(u,v) . \end{aligned}$$

The LPA system is the above plus an additional equation for the local variable:

$$\begin{aligned} \frac{\partial u_L}{\partial t}&= f(u_L,v) . \end{aligned}$$

Suppose the PDE system has a homogeneous steady state (HSS) $(u_*,v_*)$. The Jacobian of the well-mixed system and LPA system at the corresponding equilibrium is given by

$$\begin{aligned} J_{WM} = \begin{bmatrix} \partial _u f &{} \partial _v f \\ \partial _u g &{} \partial _v g \end{bmatrix}, \quad J_{LPA} = \begin{bmatrix} \partial _u f &{} \partial _v f &{} 0\\ \partial _u g &{} \partial _v g &{} 0 \\ 0 &{} \partial _v g &{} \partial _u f \\ \end{bmatrix} = \begin{bmatrix} J_{WM} &{} 0 \\ * &{} \partial _u f \end{bmatrix} , \end{aligned}$$

where the partial derivatives are understood to be evaluated at the HSS, and $*$ denote entries that are unimportant for later analysis. Notice that eigenvalues of $J_{LPA}$ are the two eigenvalues of $J_{WM}$, plus $\partial _u f$. This is due to $J_{LPA}$ being block-lower-triangular. We will show that saying the HSS is Turing-unstable in the limit $\delta \rightarrow 0$ is equivalent to saying it is a stable equilibrium for WM but unstable for LPA.

Define the relevant matrices for Turing analysis:

$$\begin{aligned} M(q^2) = J_{WM} - Dq^2, \quad D = \begin{bmatrix} \delta &{} 0 \\ 0 &{} 1 \end{bmatrix} \xrightarrow {\delta \rightarrow 0} \begin{bmatrix} 0 &{} 0 \\ 0 &{} 1 \end{bmatrix} \end{aligned}$$

Suppose the HSS satisfy the condition for Turing instability, which has three conditions (see, for example, (Edelstein-Keshet 1988, Ch. 11.4)):

5a $$\begin{aligned} {{\,\mathrm{Tr}\,}}(J_{WM})&<0 , \end{aligned}$$

5b $$\begin{aligned} \det (J_{WM})&>0 , \end{aligned}$$

5c $$\begin{aligned} \det (M(q^2))&<0 \text { for some } q^2>0 \,. \end{aligned}$$

Conditions (5a), (5b) are equivalent to saying that the HSS is a stable equilibrium for WM. Next, in the limit of $\delta \rightarrow 0$, we compute

$$\begin{aligned} \det (M) = \det (J_{WM} - Dq^2) = \det \left( J_{WM} - \begin{bmatrix} 0 &{} 0 \\ 0 &{} q^2 \end{bmatrix} \right) = \det (J_{WM}) - q^2 \partial _u f \end{aligned}$$

Notice that by setting $\delta = 0$, this equation is linear in $q^2$ instead of quadratic. This means (5c) is equivalent to $\partial _u f >0$ . But since $\partial _u f$ is an eigenvalue for $J_{LPA}$, this means the HSS is unstable in the LPA system.

Conversely, suppose that the HSS is stable in WM and unstable in LPA. This means the eigenvalues of $J_{WM}$ all have negative real part, and $\partial _u f >0$, which as shown above is equivalent to Turing-unstable.

Compared to Turing analysis, LPA has several advantages and disadvantages. LPA is essentially a “zeroth-order” expansion in $\delta$, so it is only valid in the limit of $\delta \rightarrow 0$ and offers no information on the effect of $\delta >0$, as opposed to Turing analysis. However, in this limit, LPA contains the Turing stability of the system. In particular, LPA-unstable is the same as Turing-unstable, whereas LPA-polarizable and LPA-stable regimes are Turing-stable. This follows the analysis above, and also Mata et al. (2013); Holmes (2014). This correspondence is illustrated in Fig. 23, where we show how the LPA regimes from Fig. 2b lines up with Turing regimes.

In pattern-forming regimes, LPA does not help predict the exact form of the pattern. This is most relevant to the actin feedback model, when there are many different possible patterns and a large number of parameter regimes. Turing analysis also cannot predict the final pattern, but it does allow us to predict the initial precursor pattern that forms and exists only for small *t*. In Fig. 24, we simulated the non-conservative model with parameters chosen such that only a single wave number is unstable. This results in a periodic, shallow precursor pattern which has the exact frequency as the unstable wave number.

As the system continue to evolve, once a peak of the precursor pattern reaches a certain amplitude, it very rapidly grows to the full size of the final pattern while suppressing nearby peaks. Other precursor peaks farther away from the grown one might survive longer and eventually transition to full size, or be suppressed by another nearby peak which has transitioned sooner. These non-linear interactions cannot be captured by Turing analysis.

In the special case that a static, periodic pattern forms, such as in the non-conservative model, the “minimal patch size” idea from Painter and Hillen (2011), which is based on Turing analysis, can give an upper bound on the number of periods the pattern can have.

## Appendix: Methods

Bifurcation plots were produced with XPPAUT (Ermentrout 2002; Doedel 1981) and Matcont (Dhooge et al. 2003). PDEs in 1D were solved using finite difference methods with Crank—Nicolson time stepping in Matlab with $\varDelta x= 0.005, \varDelta t= 0.0002$. Plots were produced with Matlab. PDEs in fixed 2D domain were solved using the FEniCS (Alnaes et al. 2015) package in Python, plots and movies produced by Paraview. The codes for both are published at https://github.com/liuyue002/Wave-pinning-model.

## Appendix: Cellular Potts model simulations

In the CPM, a biological cell is represented by a set of contiguous lattice site in 2D (or 3D) all assigned an index $\sigma$. (For a single cell, the index is 1 and the surrounding medium is given an index of 0.) Here we focused on a 2D CPM model cell, representing a top-down view of a “biological cell” attached to a flat surface. We use the classic Hamiltonian

6 $$\begin{aligned} H=\lambda _a(A-a)^2 +\lambda _p (P-p)^2 + J P. \end{aligned}$$

Here the three terms represent the energetic cost for change of area *A* (cell contraction/expansion) away from the preferred “rest area” *a*, a cost for elongation or shortening of the cell perimeter *P* away from a preferred “rest perimeter” *p*, and a term that describes an interfacial energy associated with the cell-medium interface. The weighting factors, $\lambda _a, \lambda _p, J$ are adjusted to set the relative importance of the various energy terms.

The perimeter *P* is approximated as in Magno et al. (2015). For each cell site, we calculate the number of lattice sites within a certain radius *r* (here $r=3$) in contact with the medium. Then we take the sum over all boundary sites and rescale by $\xi (r)$ (here $\xi (r)=18$) to obtain a perimeter approximation:

7 $$\begin{aligned} P = \frac{1}{\xi (r)} \sum _{x : \sigma (x)=1} \; \; \; \sum _{y : \lbrace |x-y|^2<r^2 \wedge \sigma (y)=0 \rbrace } 1 \end{aligned}$$

At each Monte Carlo Step (MCS) in the simulation, points along the cell edge may protrude or retract with some probability. Formally, this is achieved by *N* so-called “copy attempts”. A copy attempt consists of selecting a random site (“source”) on the lattice and copying the index into a random neighbouring site (“target”) from its Moore neighbourhood. The change/copy is accepted with probability

8 $$\begin{aligned} P(\varDelta H)= {\left\{ \begin{array}{ll} 1 &{} \text {if}\;\varDelta H + H_0 <0, \\ e^{-(\varDelta H+ H_0)/T} &{} \text {if}\;\varDelta H + H_0\ge 0. \end{array}\right. } \end{aligned}$$

Here $T\ge 0$ is denoted a cellular “temperature” and sets the intensity of random edge fluctuations. $H_0$ is a yield energy (force) to be overcome.

We start with a circular cell (12000 pixels). Assigning a nondimensional size $\varDelta x = 0.005$ to each pixel implies that cell area is 0.3. We rescaled the parameters $\eta ,k,\gamma ,\alpha ,\theta ,k_n,k_s$ with $L^2$, where $L=10$. CPM parameters *a*, *p* are in terms of pixels (see figure captions).

The initial conditions are $v=2.5, u=0$ and $F=0$ everywhere. We introduce a randomly placed circular spot (radius 3 pixels) of $u=5$ to represent an initial random burst of active GTPase. Then every 100 MCS we add another randomly placed spot of elevated GTPase $u=u + 15$ (higher than the initial burst to prevent decay) to depict stochastic bursts of GTPase activation in the cell.

For the model without F-actin, we have slightly different initial GTPase fields: $v = 1.1113, u=0.3333$, except in the upper left corner of the cell, with an area of about 1/9 of the cell, we set $u = 0.5454$.

After every MCS we solve the RD equations for 0.001s, using $dt = 10^{-6}$, so 1000 iterations per MCS. Within a MCS, after every accepted membrane extension or retraction we update the GTPase fields as follows. After an extension we set *u*(target)=*u*(source) and subsequently rescale the level of *u* throughout the cell to conserve mass. After retraction we set u(target)=0 and then u(target) is equally distributed throughout the whole cell. The same operations are carried out for *v*. For F-actin, we do the same but do not redistribute, i.e. *F*(target)=*F*(source) for extensions and *F*(target)=0 for retractions.
